# Isolation and Characterization of Two Novel Colorectal Cancer Cell Lines, Containing a Subpopulation with Potential Stem-Like Properties: Treatment Options by MYC/NMYC Inhibition

**DOI:** 10.3390/cancers12092582

**Published:** 2020-09-10

**Authors:** Jan Schulte am Esch, Beatrice Ariane Windmöller, Johannes Hanewinkel, Jonathan Storm, Christine Förster, Ludwig Wilkens, Martin Krüger, Barbara Kaltschmidt, Christian Kaltschmidt

**Affiliations:** 1 Department of General and Visceral Surgery, Protestant Hospital of Bethel Foundation, 33611 Bielefeld, Germany; Jan.SchulteamEsch@evkb.de; 2 Forschungsverbund BioMedizin Bielefeld (FBMB), 33611 Bielefeld, Germany; jonathan.storm@uni-bielefeld.de (J.S.); christine.foerster@krh.eu (C.F.); ludwig.wilkens@krh.eu (L.W.); Martin.Krueger@evkb.de (M.K.); barbara.kaltschmidt@uni-bielefeld.de (B.K.); C.Kaltschmidt@uni-bielefeld.de (C.K.); 3 Department of Cell Biology, University of Bielefeld, 33611 Bielefeld, Germany; johannes-hanewinkel@hotmail.de; 4 Institute of Pathology, KRH Hospital Nordstadt, affiliated with the Protestant Hospital of Bethel Foundation, 30167 Hannover, Germany; 5 Department of Internal Medicine and Gastroenterology, Protestant Hospital of Bethel Foundation, 33611 Bielefeld, Germany; 6 Molecular Neurobiology, University of Bielefeld, 33615 Bielefeld, Germany

**Keywords:** colorectal cancer stem cells, NMYC, MYC, ATRA, EMT, KJ-Pyr-9, rectal neuroendocrine carcinoma, colorectal adenocarcinoma

## Abstract

**Simple Summary:**

The aim of this study was to gain a better understanding of cancer stem cells, which are a small subpopulation of tumor cells with high plasticity driving tumor growth and metastasis. Here we isolated two novel colorectal cancer cell lines originating from a rectal neuroendocrine carcinoma and a colorectal adenocarcinoma, depicting stem-like properties. These in vitro models offer the possibility to evaluate pathophysiological mechanisms in order to develop tailored therapeutic strategies for distinct colorectal malignancies. Investigations revealed gene copy number gain of the N-myc proto-oncogene for both. Accordingly, inhibition of the protein–protein interaction of myc and N-myc proto-oncogenes with the myc-associated factor X utilizing small molecule KJ-Pyr-9, exhibited a significant reduction in survival of both cell lines by the induction of apoptosis. Consequently, the blockage of these interactions may serve as a possible treatment strategy for colorectal cancer cell lines with gene copy number gain of the N-myc proto-oncogene.

**Abstract:**

Cancer stem cells (CSC) are crucial mediators of cancer relapse. Here, we isolated two primary human colorectal cancer cell lines derived from a rectal neuroendocrine carcinoma (BKZ-2) and a colorectal adenocarcinoma (BKZ-3), both containing subpopulations with potential stem-like properties. Protein expression of CSC-markers prominin-1 and CD44 antigen was significantly higher for BKZ-2 and BKZ-3 in comparison to well-established colon carcinoma cell lines. High sphere-formation capacity further confirmed the existence of a subpopulation with potential stem-like phenotype. Epithelial–mesenchymal transition markers as well as immune checkpoint ligands were expressed more pronounced in BKZ-2. Both cell populations demonstrated N-myc proto-oncogene (*NMYC*) copy number gain. Myc proto-oncogene (MYC)/NMYC activity inhibitor all-trans retinoic acid (ATRA) significantly reduced the number of tumor spheres for both and the volume of BKZ-2 spheres. In contrast, the sphere volume of ATRA-treated BKZ-3 was increased, and only BKZ-2 cell proliferation was reduced in monolayer culture. Treatment with KJ-Pyr-9, a specific inhibitor of MYC/NMYC-myc-associated factor X interaction, decreased survival by the induction of apoptosis of both. In summary, here, we present the novel colorectal cancer cell lines BKZ-2 and BKZ-3 as promising cellular in vitro models for colorectal carcinomas and identify the MYC/NMYC molecular pathway involved in CSC-induced carcinogenesis with relevant therapeutic potential.

## 1. Introduction

Colorectal cancer (CRC) is the third most common cancer worldwide and second most common cause of cancer-related mortality, with 1.8 million new cases and approximately 861,000 deaths in 2018 as reported by the World Health Organization (WHO) GLOBOCON database [1]. CRC comprises the development of malignancies in the colon (72%) and the rectum (28%) and is the most common malignancy in the gastrointestinal tract [2], with 90% of all CRCs being an adenocarcinoma (AC) originating from epithelial mucosa cells [3]. A comparatively rare and poorly understood subtype of CRC is the neuroendocrine carcinoma (NEC), representing a subgroup of neuroendocrine neoplasms comprised of poorly-differentiated neoplasms originating from epithelial cells of the endocrine or nervous system [4]. According to the WHO classification of 2010, colorectal gastro-entero-pancreatic neuroendocrine-carcinoma (GEP-NEC) is a rare and aggressive variant of neuroendocrine tumors (NET) as well as CRC with more than one third having metastatic disease at the time of diagnosis [5,6]. 

To evaluate pathophysiological mechanisms and therapeutic strategies for CRCs, preclinical models were developed based on stable cell lines. However, latter are scarce especially for GEP-NECs and were mainly established from metastatic spread [7,8,9,10,11]. Although some established cellular model lines were derived from primary native tumor tissue [7,8,12], such cell lines still lack cancer stem cell characteristics. Cancer stem cells (CSC) are a small subpopulation of tumor cells of high plasticity driving tumor growth, repopulation after injury, and metastasis in a broad range of solid tumors including CRC [13,14]. Next to mediating invasiveness and resistance to chemotherapy, CSC also use immune system escape and are thus crucial mediators of cancer relapse. Next to in vitro propagation as spheres [14,15,16,17], cancer stem cell markers like prominin-1 (CD133) and CD44 antigen (CD44) are frequently used to identify cancer stem cells in CRC [3,6]. Important regulators of cancer stem cell characteristics particularly include the members of the MYC transcription factor family, consisting of L-, N-, and CMYC. In colorectal cancer stem cells (CCSC) the myc proto-oncogene protein (MYC) was reported to be consistently overexpressed, contributing to self-renewal and pluripotency as well as drug resistance [18,19,20]. Moreover, the overexpression of N-myc proto-oncogene protein (NMYC) was detected for different tumor types, most notably for cancers of neural and neuroendocrine origin [21], making the MYC-family a highly promising therapeutic target. 

Next to the influence of the MYC-family to the tumor development, recent studies showed a direct influence of MYC on the formation of metastasis by colon cancer cells in vivo [22]. Here, the authors reported that the drug Astaxanthin increases the expression of micro ribonucleic acid (miRNA)-29a-3p and miRNA-200a by the transcriptional repression of *MYC*. This repression leads to the abrogation of their downstream target genes matrix metallopeptidase 2 and zinc finger E-box binding homeobox 1 (*ZEB1*), and consequently to the suppression of epithelial–mesenchymal transition (EMT) and metastasis [22]. The process of EMT, during which epithelial cells acquire fibroblast-like properties and show reduced intercellular adhesion and increased motility, allows the tumor cells to metastasize and establish secondary tumors at distant sites [23,24]. Additionally, Xu and coworkers discovered that cells undergoing EMT acquire stem cell-like characteristics, thus providing a further link between EMT and stem cell pathways, making EMT to a possible therapeutic target, too [25]. Differentiation therapy using retinoic acids, especially all-trans retinoic acid (ATRA), showed clinical potential for different cancer types, including breast cancer [26], neuroblastoma [27], glioblastoma [28], and CRC [29]. However, ATRA is only clinically used for the treatment of acute promyelocytic leukemia [30]. In CRC cell lines treatment with ATRA led to decreased proliferation, sphere formation and aldehyde dehydrogenase (ALDH) + CSC-population size by inducing neuroendocrine differentiation [29]. Moreover, Shi and colleagues found evidence that ATRA reverses EMT in chemotherapy resistant CRC cell lines [31]. Furthermore, a study with *NMYC*-amplified neuroblastoma cells could show that a combined treatment of ATRA and a peptide derived from tenascin-C induced differentiation and led to a decrease of NMYC protein [27], highlighting the involvement of ATRA in NMYC signaling.

In the present study, we established the first primary rectal GEP-NEC cell line named BKZ-2, as well as a primary colonic AC cell line named BKZ-3 enriched for a subset of cells with markers of stemness and EMT as a pre-clinical model system. Beyond the validation of these cell lines having a subpopulation of cells with potential stem-like properties, we detected in each a genetic amplification of the oncogene *NMYC*. Although MYC/NMYC inhibition using KJ-Pyr-9 led to a significant decrease of survival of both cell lines, KJ-Pyr-9-treated BKZ-3 cells revealed a higher survival rate than BKZ-2. Additionally, we show that ATRA-treatment of BKZ-2 cells decreased sphere volume as well as total cell mass and seems to induce differentiation, as well as it decreased proliferation of BKZ-2 in monolayer. In contrast, ATRA-treatment of BKZ-3 led to an increase in sphere volume with no alteration in total cell mass or proliferation in monolayer. Next to our findings that targeting NMYC could have therapeutic activity in CRC patients, the establishment of those primary cell lines also highlights the differences in CCSC. Moreover, the isolation of BKZ-2 and BKZ-3 offers the possibility to evaluate further pathophysiological mechanisms systematically in order to develop tailored therapeutic strategies for distinct colorectal malignancies.

## 2. Results

### 2.1. Tumor Characterization

**Figure 1:**
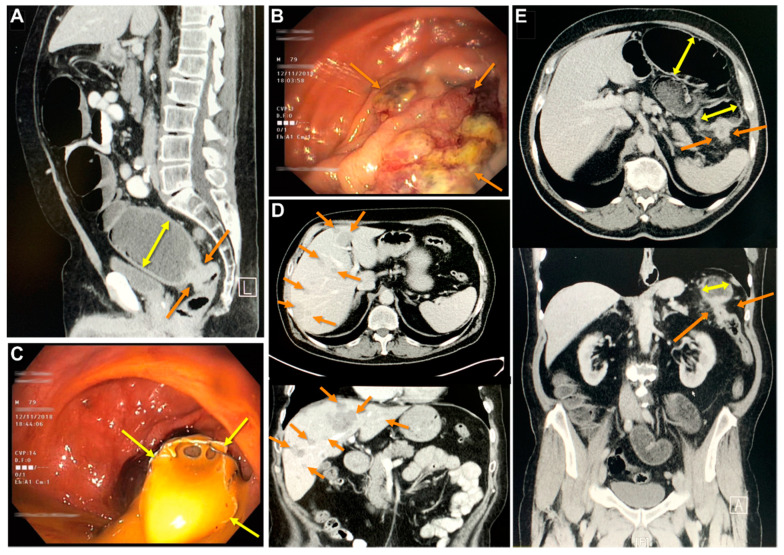
Clinical imaging derived from the two donors of the colorectal cancer cell lines. Patient BKZ-2: (**A**) Computerized tomography (CT)-scan with demonstration of a rectal stenotic mass (orange arrows) with pre-stenotic obstructed bowel (yellow double arrow). Endoscopic appearance of carcinoma BKZ-2 (**B**) with intestinal discharge following (**C**) endoscopic bowel stenting of the neoplastic stenosis. (**D**) Staging CT-scan visualizing hepatic metastases (orange arrows). Patient BKZ-3: (**E**) CT-scan indicating the neoplastic mass of the left colon (orange arrows) with pre-stenotic obstructed bowel (yellow double arrows).

**Figure 2:**
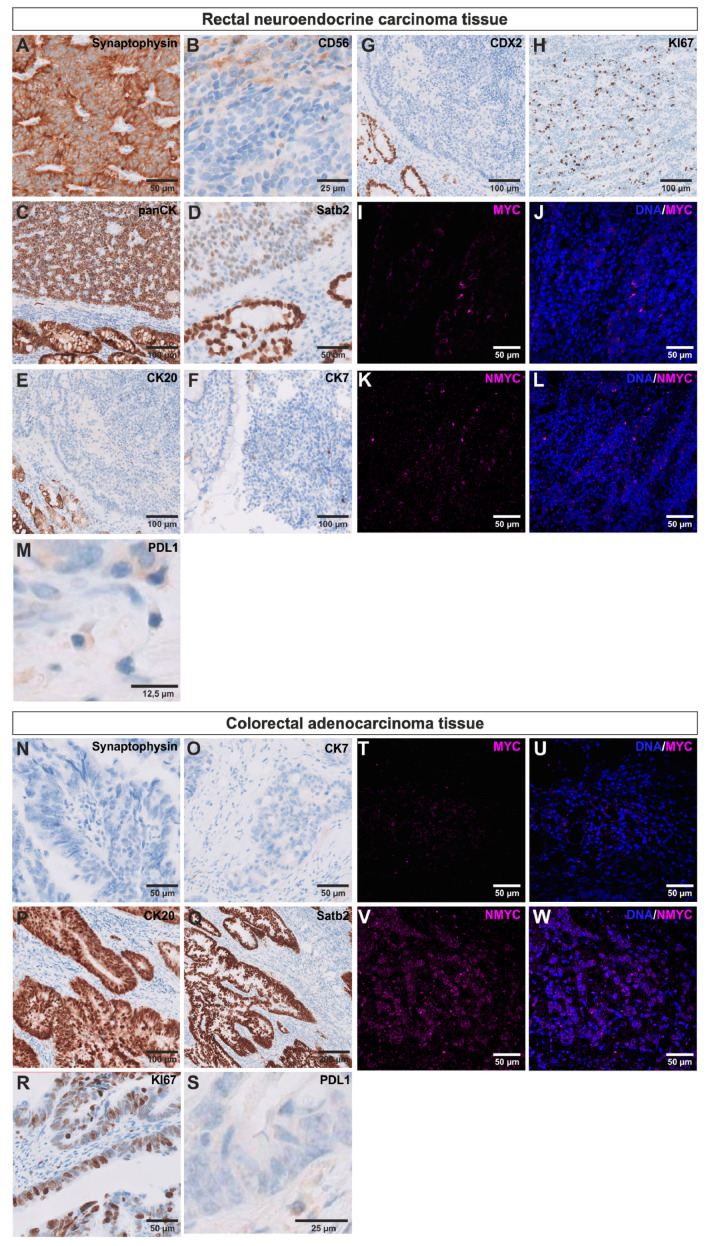
Immunohistochemical characterization of the primary rectal large cell neuroendocrine carcinoma (NEC) and the colorectal adenocarcinoma (AC). NEC tissue was tested positive for (**A**) Synaptophysin, (**B**) neural cell adhesion molecule (CD56), (**C**) epithelial marker pan-cytokeratin (panCK) and (**D**) special AT-rich sequence-binding protein 2 (SATB2), but was negative for (**E**) cytokeratin 20 (CK20) and (**F**) cytokeratin 7 (CK7). Moreover, immunohistological staining for (**G**) the intestinal differentiation marker homeobox protein CDX2 was negative. (**H**) Staining for the proliferation marker protein Ki-67 (KI67) revealed 25% positive cells. Further immunohistochemical stainings of the NEC tissue displayed positivity for the (**I**/**J**) myc proto-oncogene protein (MYC) and (**K**/**L**) N-myc proto-oncogene protein (NMYC). (**M**) Immunohistochemical staining for programed death ligand 1 (PDL1) revealed only slight expression with 2% of vital tumor cells being positive. AC tissue was tested negative for neuroendocrine marker (**N**) Synaptophysin and (**O**) CK7, but was positive for (**P**) CK20 and (**Q**) SATB2. AC revealed (**R**) 50% KI67 highly positive cells and 25% cells with moderate KI67 expression. (**S**) Immunohistochemical characterization of PDL1 expression displayed 0% positive vital tumor cells, but revealed positivity for both (**T**/**U**) MYC and (**V**/**W**) NMYC in the AC tissue.

An endoscopically retrieved bioptic sample of the primary tumor of patient 1 (Figure 1A–D) revealed the pathological diagnosis of a rectal NET with G3 differentiation and 25% proliferation marker protein Ki-67 (KI67) expression (Figure 2H). It was 100% positive for epithelial marker pan-cytokeratin (panCK), 40% positive for special AT-rich sequence-binding protein 2 (SATB2), 10% positive for neural cell adhesion molecule (CD56) and 100% positive for Synaptophysin (Figure 2A–D) characterizing the colorectal tumor as large cell GEP-NEC according to the WHO-classification of 2010. It was tested negative for cytokeratin 20 (CK20), cytokeratin 7 (CK7) and homeobox protein CDX-2 (CDX2) (Figure 2E–G). Next to the immunopathological analysis of the tissue, we performed an immunohistochemical staining for the proto-oncogenes MYC and NMYC, which were both positive (Figure 2I–L). Analysis of the expression of the immune checkpoint ligand programed death ligand 1 (PDL1) was done according to international standards by calculating combined positive score (CPS) and tumor proportion score (TPS), as described in the material and methods section (Formulas (3) and (4)). Quantification revealed 2% of PDL1 positive non-necrotic tumor cells. Analysis of the percentage of PDL1 positive vital (non-necrotic) tumor cells, lymphocytes and macrophages were done by calculating CPS and revealed a score of 2. Quantification of PDL1 positive lymphocytes, macrophages, dendritic cells and granulocytes per tumor area displayed an immune cell score (IC) of 1 (Figure 2M, Formula (5)). There was no micro-satellite-instability nor positivity for human epidermal growth factor receptor (EGFR) 2. Kirsten rat sarcoma 2 viral oncogene homolog/neuroblastoma rat sarcoma viral oncogene homolog evaluation revealed a wild type. 

Histopathological examination of a bioptic sample retrieved from the primary tumor of patient 2 (Figure 1E) revealed the diagnosis of a colorectal AC. Carcinoma tissue was 100% positive for SATB2, 95% positive for CK20 and positive for KI67 with 50% being highly positive and 25% expressing moderate levels of KI67 (Figure 2P–R). Analysis of the expression of PDL1 revealed 0% of PDL1 positive vital tumor cells. However, CPS was 3 for the adenocarcinoma tissue and IC was 1 (Figure 2S). Further, it was tested negative for CK7 and Synaptophysin, characterizing the tumor as a colorectal AC (Figure 2N,O). Analysis of the two oncogenes MYC and NMYC revealed positivity of the tissue for both (Figure 2T–W). 

### 2.2. BKZ-2 and BKZ-3 Demonstrate Characteristics of Stemness and EMT–Relation to Immune Response Involved Targets

**Figure 3:**
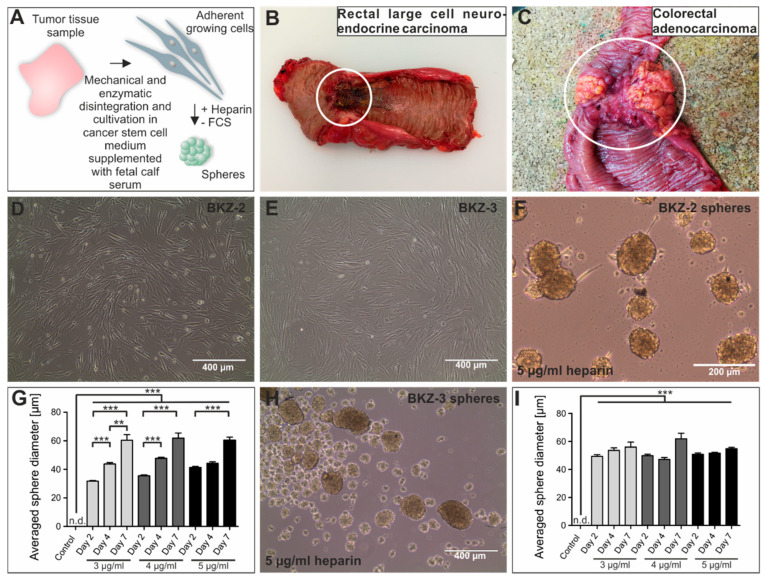
Successful isolation of the rectal large cell neuroendocrine carcinoma (NEC)-derived cancer cell line BKZ-2 and the colorectal adenocarcinoma (AC)-derived cancer cell line BKZ-3. (**A**) For the isolation of those cell lines that contain a subpopulation of cells with potential stem-like properties a tissue sample of either the (**B**) rectal large cell NEC or the (**C**) colorectal AC was obtained, mechanically and enzymatically disintegrated, and cultivated in CSC medium supplemented with fetal calf serum (FCS), leading to (**D**/**E**) adherent growing cells. (**F**/**H**) Cultivation of the cells with the addition of heparin and in the absence of FCS led to the formation of spheres, further validating stem-like properties of BKZ-2 and BKZ-3. (**G**/**I**) Quantification of the averaged sphere diameter showed a significant increase after the addition of heparin in comparison to the control for BKZ-2 and BKZ-3, regardless of the tested heparin concentrations. Moreover, BKZ-2 showed a continuous growth of the spheres over a time-period of one week. Non-parametric Kruskal-Wallis test (*p* ≤ 0.05), followed by Dunn’s Multiple Comparison post-hoc test. *n* = 5, *** *p* ≤ 0.001, ** *p* ≤ 0.01. Mean ± standard error of the mean (SEM). n.d. = not detectable.

**Figure 4:**
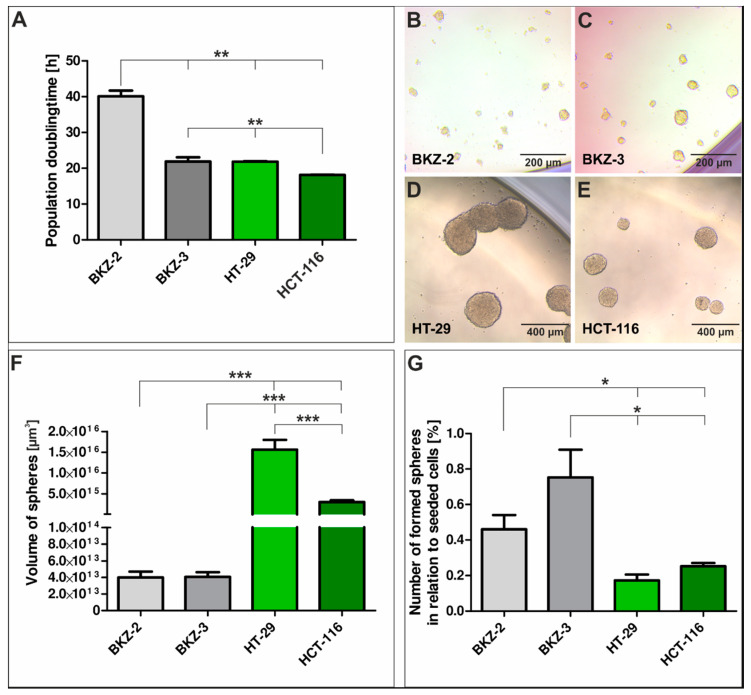
BKZ-2 and BKZ-3 reveal higher population doubling times and formed higher numbers of spheres in comparison to HT-29 and HCT-116. (**A**) Quantification of the population doubling times of the newly isolated colorectal cancer cell lines BKZ-2 and BKZ-3 as well as the common colon adenocarcinoma cell line HT-29 and colon carcinoma cell line HCT-116 revealed a significantly higher population doubling time for BKZ-2 in comparison to BKZ-3, HT-29 and HCT-116. Moreover, BKZ-3 and HT-29 displayed a significantly higher population doubling time when compared with HCT-116. (**B**–**E**) All cell populations formed spheres when 5000 cells per 200 μL cancer stem cell (CSC) medium containing 4 μg/mL heparin were cultured in low adhesion 96 well-plates. Quantification of the (**F**) volume of spheres formed by each cell line showed a significantly higher volume for HT-29 and HCT-116 when compared to BKZ-2 and BKZ-3. Moreover, sphere volume of HT-29 was significantly higher in comparison to HCT-116. Further quantification concerning (**G**) the number of formed spheres in relation to seeded cells revealed significantly less percent spheres for HT-29 and HCT-116 in comparison to BKZ-2 and BKZ-3. Non-parametric Mann-Whitney-test (*p* ≤ 0.05). *n* ≤ 3, *** *p* ≤ 0.001, ** *p* ≤ 0.01, * *p* ≤ 0.05. Mean ± SEM (standard error of the mean).

We established colorectal cancer cell lines BKZ-2 and BKZ-3 derived from primarily resected native tumor tissue (Figure 3A–C) by mechanical and enzymatic disintegration of the tissue sample. Subsequent cultivation within Dulbecco’s Modified Eagle’s Medium/Ham’s -F12 supplemented with epidermal growth factor (EGF), fibroblast growth factor 2 (FGF-2), B27 and 10% fetal calf serum (FCS) led to an adherently growing cell culture for both tumor tissue samples (Figure 3D,E). Initial culture conditions were designed to obtain subpopulation of CSC from native tumor tissue. Analysis of serine/threonine-protein kinase B-raf (*BRAF*) gene mutations displayed wild type alleles of *BRAF* for exon 11 and 15 for both parental tumor tissue and isolated BKZ-2 and BKZ-3 cells. To confirm the stem-like nature of BKZ-2 and BKZ-3, we tested the sphere formation capacity of both cell populations. Both BKZ-2 and BKZ-3 formed spheres under serum-free conditions and supplementation with heparin (Figure 3F,H). Quantification of the averaged sphere diameter of BKZ-2 and BKZ-3 revealed a significant difference of the sphere-formation capacity for all three different heparin concentrations and time points in comparison to the control. Moreover, the increase in sphere diameter of BKZ-2 was significant with a peak value of 61.9 μm (±0.35) in the approach with 4 μg/mL heparin after 7 days of culture. However, the increase in sphere diameter of BKZ-3 cells was not significant, although there was also a tendency to form larger spheres over time with the highest value of sphere diameter of 61.9 μm (±3.95) after the addition of 4 μg/mL heparin and cultivation for one week (Figure 3G,I). Quantification of the population doubling time of BKZ-2 and BKZ-3 in comparison to the established colon adenocarcinoma cell line HT-29 and colon carcinoma cell line HCT-116, revealed a significantly higher (*p* ≤ 0.01) population doubling time for BKZ-2 with 40.12 h (±1.56) in comparison to BKZ-3 with 21.88 h (±1.19). Furthermore, HT-29 displayed a population doubling time of 21.87 h (±0.12) and HCT-116 of 18.14 h (±0.051), which were significantly lower than the newly described cell line BKZ-2. In addition, BKZ-3 and HT-29 both displayed a significantly higher population doubling time in comparison to HCT-116 (Figure 4A, Formulas (1) and (2)). Comparison of sphere formation capacity of BKZ-2, BKZ-3, HT-29 and HCT-116, revealed a significantly higher (*p* ≤ 0.001) volume of spheres formed by HT-29 and HCT-116 when compared to BKZ-2 and BKZ-3. Moreover, HT-29 spheres displayed a significantly (*p* ≤ 0.001) higher volume in comparison to HCT-116 (Figure 4B–F, Formula (6)). Further quantification concerning the number of spheres in relation to the count of seeded cells, showed more than double amount of sphere formation rates for BKZ-2 and BKZ-3 (*p* ≤ 0.05) in comparison to HT-29 and HCT-116 (Figure 4G). 

**Figure 5:**
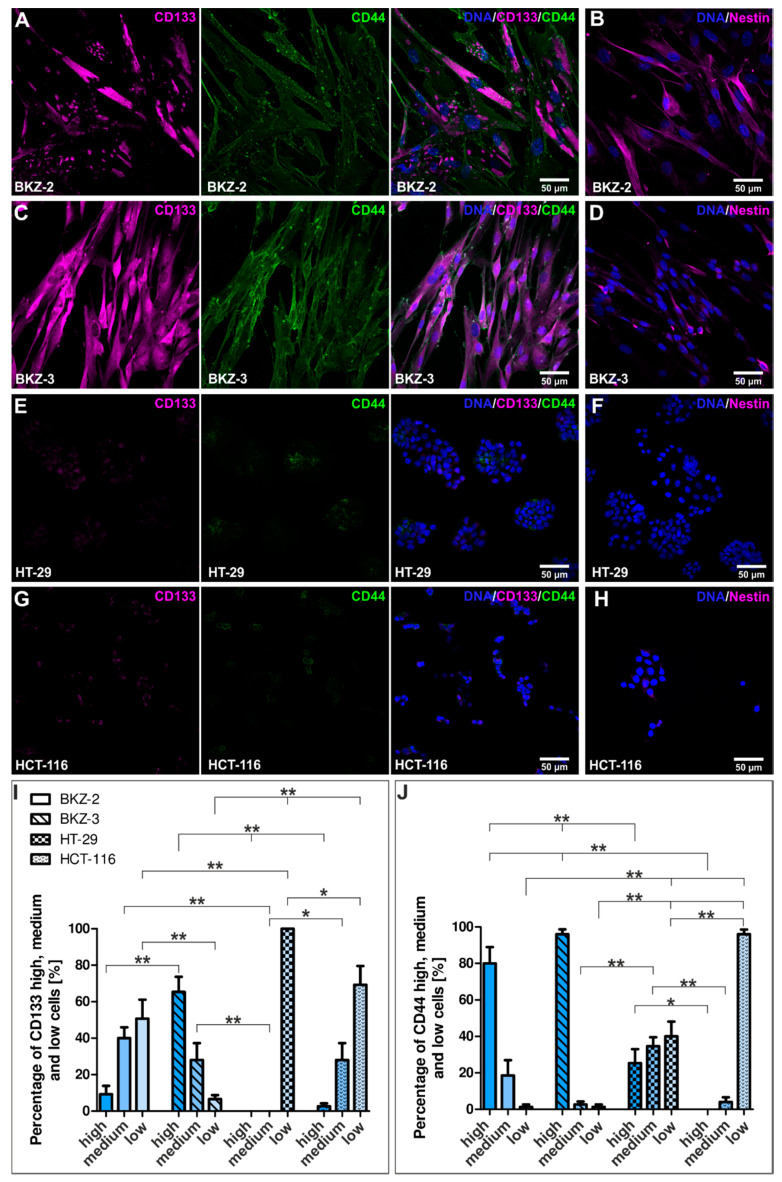
BKZ-2 and BKZ-3 express higher amounts of prominin-1 (CD133) and CD44 antigen (CD44) in comparison to HT-29 and HCT-116. Immunocytochemical analysis of BKZ-2 and BKZ-3 displayed high positivity for the cancer stem cell (CSC)-markers (**A/C**) CD133, CD44 and (**B/D**) Nestin, validating the isolation of two new cell lines that contain a subpopulation of cells with potential stem-like properties. Immunocytochemical analysis of the common colon carcinoma cell lines HT-29 and HCT-116 only displayed slight expression of the CSC-markers (**E**/**G**) CD133, CD44 and (**F**/**H**) Nestin. Quantification of the percentage of (**I**) CD133 high, medium and low cells revealed a significantly elevated amount of CD133 high BKZ-3 cells in comparison to BKZ-2, HT-29 and HCT-116. Moreover, the percentage of HT-29 and HCT-116 CD133 low cells was significantly higher when compared to BKZ-2 and BKZ-3. Quantification of (**J**) CD44 high, medium and low cells displayed for both populations a significantly higher percentage of CD44 high cells in comparison to HT-29 and HCT-116. Non-parametric Kruskal-Wallis equality-of-populations rank test (*p* ≤ 0.05) followed by Mann-Whitney test (*p* ≤ 0.05). *n* = 3, ** *p* ≤ 0.01, * *p* ≤ 0.05, ns = not significant. Mean ± SEM (standard error of the mean).

**Figure 6:**
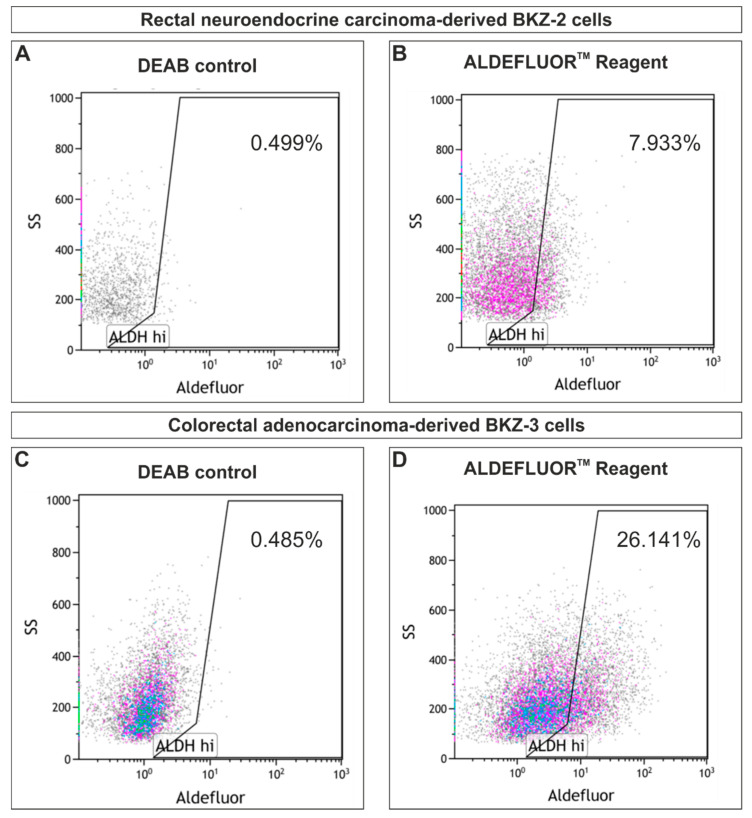
BKZ-2 and BKZ-3 both show aldehyde dehydrogenase (ALDH) activity. Flow-cytometric-analysis of ALDH activity of (**B**) BKZ-2 and (**D**) BKZ-3 revealed 7.993% ALDH high cells for BKZ-2 and 26.141% ALDH high cells for BKZ-3 in comparison to the appropriate (**A**/**C**) control with the specific ALDH inhibitor diethylaminobenzaldehyde (DEAB).

In addition to the sphere formation capacity, BKZ-2 and BKZ-3 express the prominent CSC-markers CD133, CD44 and Nestin on protein level (Figure 5A–D and Figure S3). Immunocytochemical analysis of CD133, CD44 and Nestin expression of colon adenocarcinoma cell line HT-29 and colon carcinoma cell line HCT-116 only revealed slight expression of these CSC-markers (Figure 5E–H). Quantification of the percentage of CD133 high, medium and low cells showed significantly (*p* ≤ 0.01) higher percentages of CD133 high cells for BKZ-3 cells in comparison to BKZ-2, HT-29 and HCT-116. Moreover, HT-29 and HCT-116 revealed significantly (*p* ≤ 0.01) more CD133 low cells in comparison to BKZ-3. Even though BKZ-2 cells showed significantly more CD133 low cells in comparison to BKZ-3, HT-29 revealed significantly more CD133 low cells when compared to BKZ-2. Additionally, BKZ-2 revealed more CD133 medium cells when compared to HT-29 (Figure 5I). Quantification of CD44 expressing cells revealed no difference between BKZ-2 and BKZ-3 concerning the percentage of CD44 high cells. However, both cell lines showed significantly (*p* ≤ 0.01) more CD44 high expressing cells in comparison to HT-29 and HCT-116 (Figure 5J). Thus, both HT-29 and HCT-116 revealed a tendency for higher percentages of CD133 and CD44 low expressing cells, as percentage of CD44 medium expressing cells was significantly higher in BKZ-2 and BKZ-3, too (Figure 5I,J). Further flow cytometric analysis concerning the ALDH activity of BKZ-2 and BKZ-3 showed that 7.93% of BKZ-2 and 26.14% of BKZ-3 cells are ALDH high expressing cells (Figure 6), further showing that those cell lines contain a subpopulation of cells with potential stem-like characteristics. 

**Figure 7:**
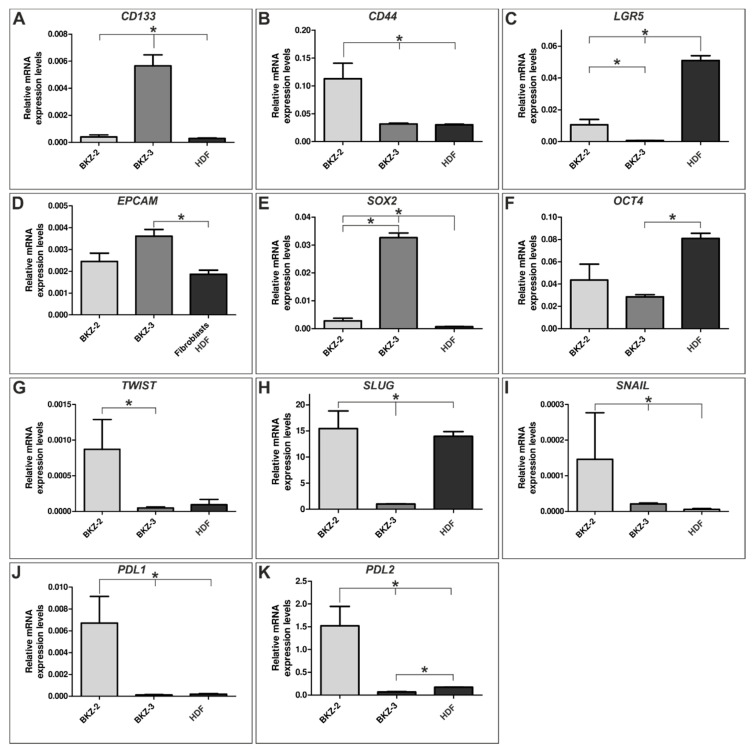
BKZ-2 and BKZ-3 show higher messenger ribonucleic acid (mRNA)-level of cancer stem cell (CSC)- and epithelial-mesenchymal-transition (EMT)-markers as well as immune checkpoint ligands in comparison to human dermal fibroblasts (HDF). Quantitative polymerase chain reaction revealed an expression of CSC-markers (**A**) prominin-1 (*CD133*), (**B**) CD44 antigen (*CD44*), (**C**) leucine rich repeat containing G protein-coupled receptor 5 *(LGR5*), (**D**) epithelial cell adhesion molecule (*EPCAM*), (**E**) SRY-box transcription factor 2 (*SOX2*) and (**F**) octamer-binding transcription factor 4 (*OCT4*) in both cell lines. Comparison of the two cell lines, demonstrated significant differences of the relative mRNA expression for *CD133*, *CD44*, *LGR5* and *SOX2*. Further analysis revealed an expression of the key transcription factors of the process of EMT (**G**) twist family bHLH transcription factor 1 (*TWIST*), (**H**) snail family transcriptional repressor 2 (*SLUG*) and (**I**) snail family transcriptional repressor 1 (*SNAIL*), with *TWIST* and *SLUG* being significantly different expressed in BKZ-2 and BKZ-3. Moreover, quantification displayed a significantly altered expression of the immune checkpoint ligands (**J**) programmed death ligand 1 (*PDL1*) and (**K**) programmed death ligand 2 (*PDL2*) in the two cell lines. Non-parametric Mann-Whitney-test (*p* ≤ 0.05). *n* = 3, * *p* ≤ 0.05. Mean ± SEM (standard error of the mean).

Further investigation concerning the transcriptional profile of BKZ-2 and BKZ-3 in comparison to human dermal fibroblasts (HDF) via quantitative polymerase chain reaction (qPCR) exhibited the expression of CSC-markers *CD133*, *CD44*, leucine rich repeat containing G protein-coupled receptor 5 (*LGR5*) and epithelial cell adhesion molecule (*EPCAM*) as well as the expression of SRY-box transcription factor 2 (*SOX2*) and octamer-binding transcription factor 4 (*OCT4*) in both BKZ-2 and BKZ-3. A statistical comparison of the transcription levels of BKZ-2, BKZ-3, and HDF revealed significant differences in the expression of *CD133* (*p* ≤ 0.05), with BKZ-3 revealing higher levels in comparison to BKZ-2 and HDF and *CD44* (*p* ≤ 0.05), with BKZ-2 showing higher expression level in comparison to BKZ-3 and HDF (Figure 7A,B). Even though, *LGR5* was significantly higher expressed in BKZ-2 when compared to BKZ-3 (*p* ≤ 0.05), both BKZ-2 and BKZ-3 show significantly lower (*p* ≤ 0.05) level in comparison to HDF (Figure 7C). Comparison of the endothelial and CSC-marker *EPCAM* revealed significantly higher (*p* ≤ 0.05) expression for BKZ-3 in comparison to HDF, but no difference between BKZ-2 and BKZ-3 (Figure 7D). *SOX2* was significantly higher (*p* ≤ 0.05) expressed in BKZ-2 and BKZ-3 in comparison to HDF, with significantly higher (*p* ≤ 0.05) expression for BKZ-3 when compared to BKZ-2 (Figure 7E). The pluripotency marker *OCT4* did not show any statistical difference between the two cell lines, but was significantly higher expressed in HDF in comparison to BKZ-3 (Figure 7F). As phenotypic stemness often coincides with a pronounced ability to proliferate, to migrate and to invade in consequence of EMT, we tested for EMT-markers such as snail family transcriptional repressor 2 (*SLUG*), snail family transcriptional repressor 1 (*SNAIL*) and twist family bHLH transcription factor 1 (*TWIST*). Although the three key transcription factors of EMT were detectable in both cell lines, the expression levels of BKZ-2 were significantly higher than the expression of BKZ-3 for *TWIST* (*p* ≤ 0.05) and especially for *SLUG* (*p* ≤ 0.05). Moreover, *SNAIL* was expressed significantly higher in BKZ-2 and BKZ-3 in comparison to HDF (Figure 7G–I). Further, we tested for immune response checkpoint related ligands *PDL1* and programmed death ligand 2 (*PDL2*). Transcriptional analysis revealed an expression of both *PDL1* and *PDL2* in BKZ-2 and BKZ-3, with *PDL2* being higher expressed than *PDL1*. Quantification displayed a significantly higher expression of *PDL1* (*p* ≤ 0.05) and *PDL2* (*p* ≤ 0.05) for BKZ-2 in comparison to BKZ-3 and HDF (Figure 7J,K).

### 2.3. BKZ-2 and BKZ-3 Feature Neuroendocrine, Neural Crest and Neuronal Characteristics

**Figure 8:**
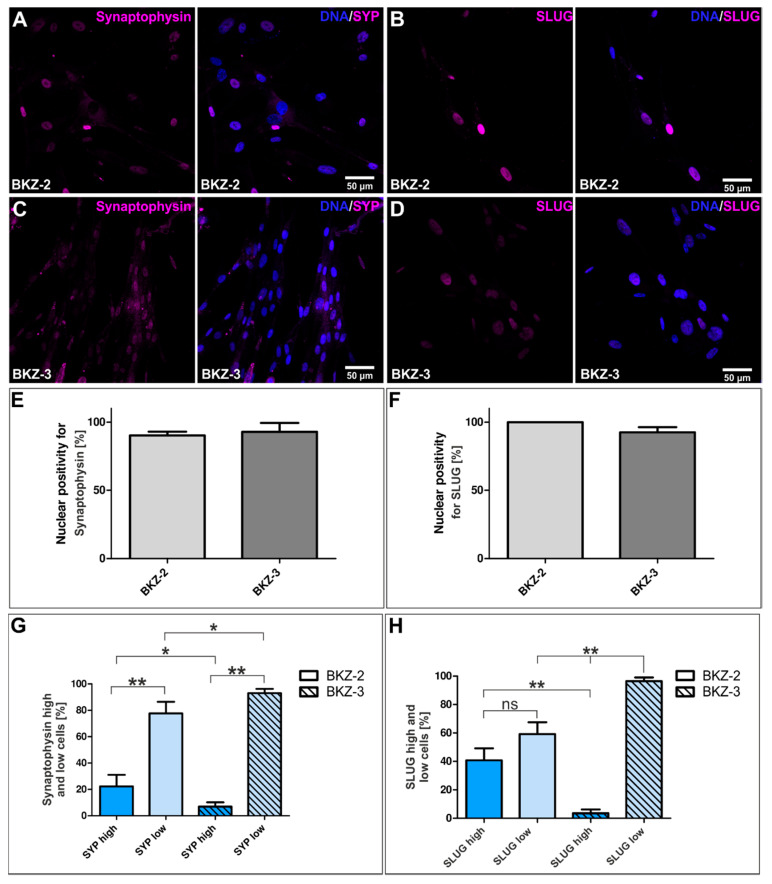
BKZ-2 cells reveal higher levels of Synaptophysin (SYP) and snail family transcriptional repressor 2 (SLUG) protein in comparison to BKZ-3 cells. Immunocytochemical stainings revealed the expression of neuroendocrine and cancer stem cell marker (**A/C**) Synaptophysin as well as the expression of (**B/D**) SLUG, one of the key transcription factors of the process of epithelial to mesenchymal transition in both populations. (**E**) Quantification of cells positive for nuclear Synaptophysin revealed a mean of 90.27% for BKZ-2 and 92.92% for BKZ-3. (**G**) Further classification in Synaptophysin high and low cells, showed a significantly higher amount of Synaptophysin low nuclei in comparison to Synaptophysin high nuclei for both BKZ-2 and BKZ-3. However, BKZ-2 revealed a significantly higher percentage of Synaptophysin high nuclei in comparison to BKZ-3. (**F**) Quantification of nuclear positivity for SLUG displayed 100% positive cells for BKZ-2 and a mean of 92.57% positive cells for BKZ-3. (**H**) Comparison of SLUG high and low cells displayed a significantly higher amount of SLUG high cells of BKZ-2 when compared to BKZ-3. Moreover, BKZ-3 cells in general showed a significantly higher percentage of SLUG low cells in comparison to the amount of SLUG high cells. Non-parametric Mann-Whitney-test (*p* ≤ 0.05). *n* = 3, ** *p* ≤ 0.01, * *p* ≤ 0.05, ns = not significant. Mean ± SEM (standard error of the mean).

In a next step, we tested whether BKZ-2 share both neuroendocrine as well as neuronal characteristics respectively as a cell line derived from a rectal carcinoma typed as a neuroendocrine carcinoma. Synaptophysin was expressed on protein level confirmed by immunocytochemistry as nuclear staining for BKZ-2 (Figure 8A). Moreover, BKZ-3 was also tested positive for Synaptophysin (Figure 8C). Quantification of the percentage of nuclear Synaptophysin positive cells revealed 90.27% (±2.73) for BKZ-2 and 92.92% (±6.45) for BKZ-3 (Figure 8E). Further quantification concerning Synaptophysin high and low nuclei displayed significantly more high nuclei (*p* ≤ 0.05) for BKZ-2 in comparison to BKZ-3. However, both cell populations revealed significantly more Synaptophysin low (*p* ≤ 0.01) nuclei in comparison to Synaptophysin high (Figure 8G). 

As neural crest characteristics are strongly related to the EMT process, we also investigated the expression of key transcription factor SLUG and neural crest and calcium binding protein S100 (S100) on protein level. Immunological staining displayed a strong expression of nuclear SLUG in both BKZ-2 and BKZ-3, representing an EMT-phenotype (Figure 8B,D). Quantification of the amount of nuclear SLUG positive cells displayed 100% positive BKZ-2 cells and 92.57 (±3.71) positive BKZ-3 cells (Figure 8F). Further quantification revealed significantly more (*p* ≤ 0.01) SLUG high cells for BKZ-2 in comparison to BKZ-3. Additionally, BKZ-3 cells showed significantly more (*p* ≤ 0.01) SLUG low cells when compared to the amount of SLUG low BKZ-2 cells and SLUG high BKZ-3 cells (Figure 8H). Moreover, S100 could be detected nuclear as well as cytoplasmatic in both cell lines (Figure S1A,E). Next, further traits of neuronal differentiation utilizing a comprehensive panel of neuronal markers were evaluated. Examined by immunocytochemistry, BKZ-2 and BKZ-3 were positive for cytosolic vesicular glutamate transporter 2 (vGLUT2), cytosolic dopamine and mainly nuclear tyrosine hydroxylase (TH), highlighting their neuronal characteristic. Moreover, the nuclear expression of TH indicates an undifferentiated phenotype, further suggesting stemness-like features of a subpopulation of BKZ-2 and BKZ-3 since TH was also detectable in the nuclei of BKZ-3 (Figure S1B–D,F–H). HDF were used as negative control for all immunocytochemical stainings (Figure S2A–J).

### 2.4. ATRA-Treatment Leads to a Contrary Switch in Growth Habits of BKZ-2 and BKZ-3

**Figure 9:**
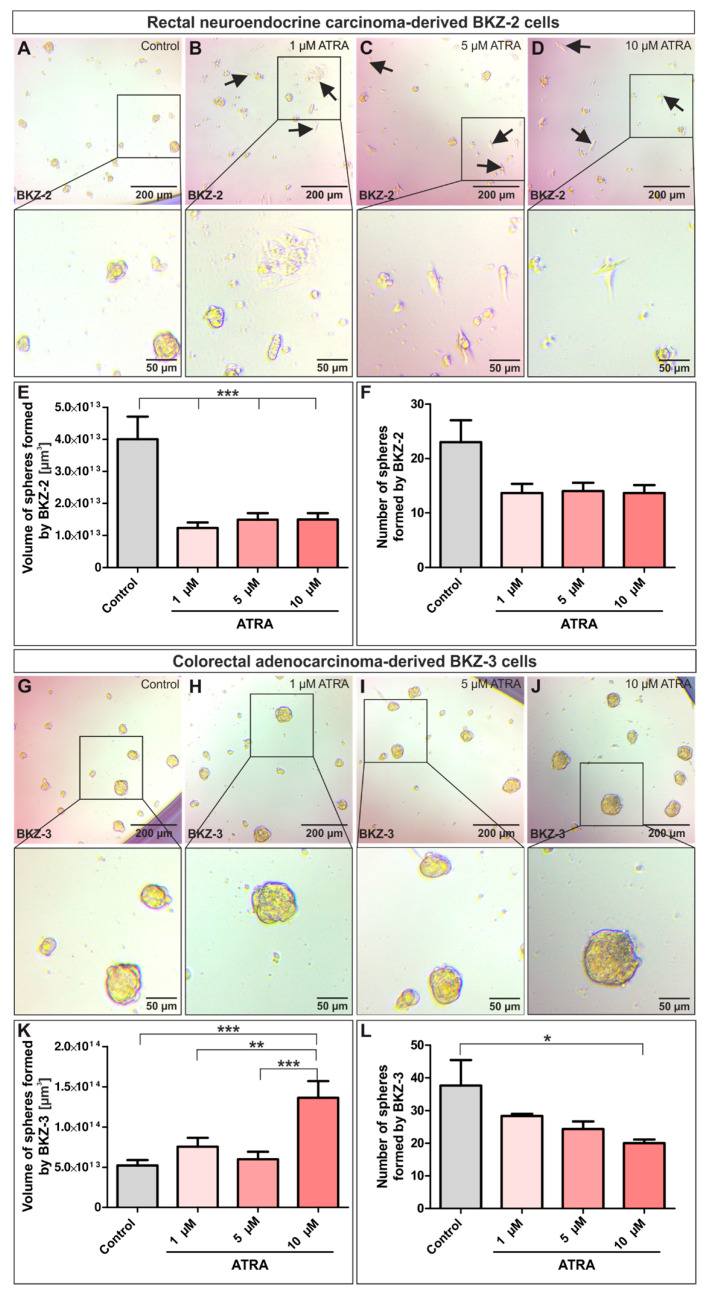
All-trans retinoic acid (ATRA) reduce number of BKZ-2 and BKZ-3 formed spheres respectively, but cause opposed effects concerning sphere volume of BKZ-2 and BKZ-3. Cells were cultured in an amount of 5000 cells per 200 μL cancer stem cell (CSC) medium containing 4 μg/mL heparin in a low adhesion 96 well-plate. Medium was supplemented with (**A**/**G**) dimethylsulfoxide, (**B**/**H**) 1 μM ATRA, (**C**/**I**) 5 μM ATRA or (**D**/**J**) 10 μM ATRA. (**A**–**D**) Representative images already display a morphological change of BKZ-2 after the cultivation with 1 μM ATRA, indicated by the adherence of the cells (arrows). Quantification of the (**E**) volume of spheres formed by BKZ-2 cells showed a significant decrease after ATRA-treatment. Further quantification concerning (**F**) the number of spheres revealed a tendency for fewer spheres after ATRA-treatment for BKZ-2. (**G**–**J**) Representative images of BKZ-3 spheres and quantification of the (**K**) volume of spheres formed by BKZ-3 cells showed a significant increase subsequent to treatment with 10 μM ATRA. Further quantification concerning the (**L**) number of spheres revealed a significant decrease of the number of spheres after the treatment with 10 μM ATRA. Non-parametric Mann-Whitney-test (*p* ≤ 0.05). *n* = 3, *** *p* ≤ 0.001, ** *p* ≤ 0.01, * *p* ≤ 0.05. Mean ± SEM (standard error of the mean).

Next we investigated the influence of ATRA on BKZ-2 and BKZ-3 cells as ATRA was shown to decrease proliferation, sphere formation and CSC population size in CRC cell lines. For this, sphere-formation capacity of BKZ-2 and BKZ-3 was analyzed after treatment with different ATRA concentrations. After five days of culture, volume, and number of spheres of BKZ-2 were quantified, revealing a significant decrease of sphere volume (*p* ≤ 0.001) after cultivation with 1, 5, or 10 μM ATRA (Figure 9E, Formula (6)). Furthermore, there was a non-significant trend of reduced number of spheres formed by BKZ-2 when co-incubated with ATRA regardless of used concentration (Figure 9F). Representative images of each treatment condition suggested the induction of a differentiation towards fibroblast-like morphology of BKZ-2 upon ATRA-treatment with cells becoming more adherent (Figure 9A–D).

**Figure 10:**
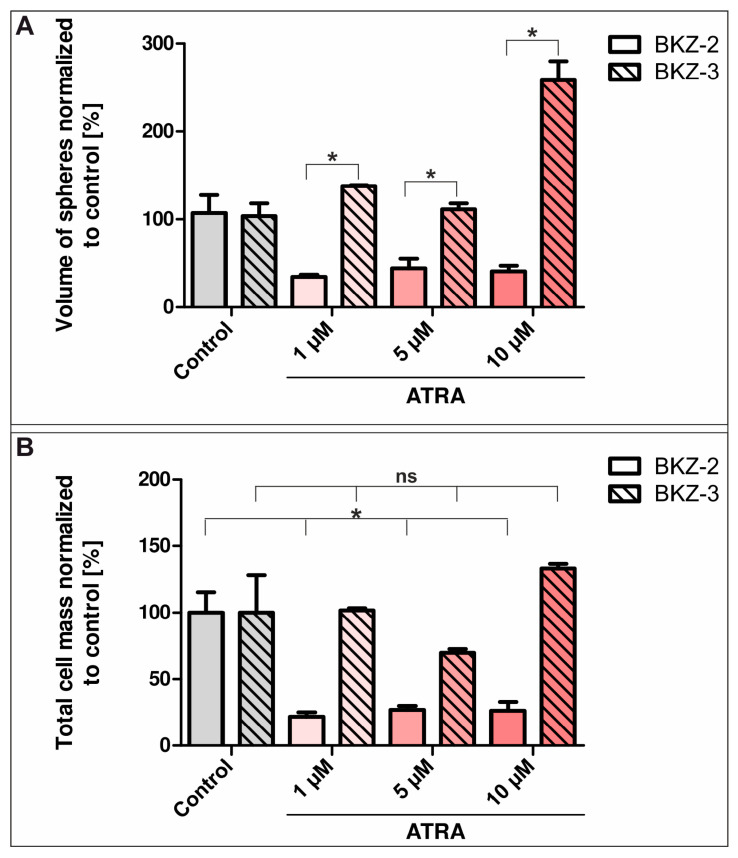
All-trans retinoic acid (ATRA)-treatment reduced total cell mass of BKZ-2 formed spheres, but does not have an effect on total cell mass of BKZ-3 formed spheres. Analysis of the quantification of the (**A**) volume of the spheres formed by the two colorectal cancer cell lines revealed a significant difference after ATRA-treatment. ATRA-treatment led to the formation of significant bigger spheres formed by BKZ-3 in comparison to BKZ-2. Further quantification of (**B**) the total cell mass revealed a significantly decreased cell mass for BKZ-2 upon ATRA stimulation, while the total cell mass of BKZ-3 was not altered. Non-parametric Mann-Whitney-test (*p* ≤ 0.05). *n* = 3, * *p* ≤ 0.05, ns = not significant. Mean ± SEM (standard error of the mean).

A corresponding experiment was performed for BKZ-3, however unexpectedly results were opposed to those for BKZ-2. Quantification revealed no significant decrease as for BKZ-2, but a significant increase in sphere volume of BKZ-3 after 10 μM ATRA-treatment (*p* ≤ 0.001) (Figure 9G–K). In contrast to the increase of sphere volume, number of spheres formed by BKZ-3 declined significantly when treated with 10 μM ATRA (*p* ≤ 0.05) (Figure 9L), suggesting a switch in the growth behavior of BKZ-3 upon ATRA stimulation. A direct comparison of the volume of formed spheres by BKZ-2 and BKZ-3 revealed a statistically significant difference between the volumes of spheres induced by ATRA stimulation (*p* ≤ 0.05). The sphere volume of BKZ-3 was significantly higher in comparison to BKZ-2 independent of ATRA concentration (Figure 10A). Additionally, calculation of the total cell mass of BKZ-2 and BKZ-3 by multiplying the mean of the volume with the mean of sphere number exhibited a significant decrease for BKZ-2 (*p* ≤ 0.05), but no alteration in cell mass for BKZ-3 upon ATRA-treatment (Figure 10B). Thus, ATRA stimulation seems to lead to a switch in growth behavior of spheres in both cell lines, however BKZ-2 cells seem to switch to a more differentiated phenotype with less total cell mass and BKZ-3 cells generate bigger but fewer spheres without any significant change in total cell mass. Next to the analysis of the influence of ATRA on sphere formation of BKZ-2 and BKZ-3, its influence on monolayer cultures was investigated. For this, BKZ-2 and BKZ-3 cells as monolayers were treated for five days with different ATRA concentrations. Afterwards, the cell count was determined utilizing Orangu^TM^ (Cell Guidance Systems, Cambridge, UK). Statistical analysis revealed a significant decrease (*p* ≤ 0.05) in cell count for BKZ-2 cells after ATRA-treatment, but not for BKZ-3 (Supplementary Figure S4A,B). Further immunocytochemical stainings for cleaved caspase 3 protein after ATRA-treatment of BKZ-2 and BKZ-3 did not showed any positivity for this apoptosis marker, suggesting an influence of ATRA on cell proliferation only (Figure S4C–J). Finally, BKZ-2 and BKZ-3 seem to behave differently in relation to ATRA, even though ATRA reduced the number of formed spheres for both cell populations.

### 2.5. NMYC Copy Number Gain Sensitize BKZ-2 and BKZ-3 for MYC/NMYC Inhibitor Induced Apoptosis

**Figure 11:**
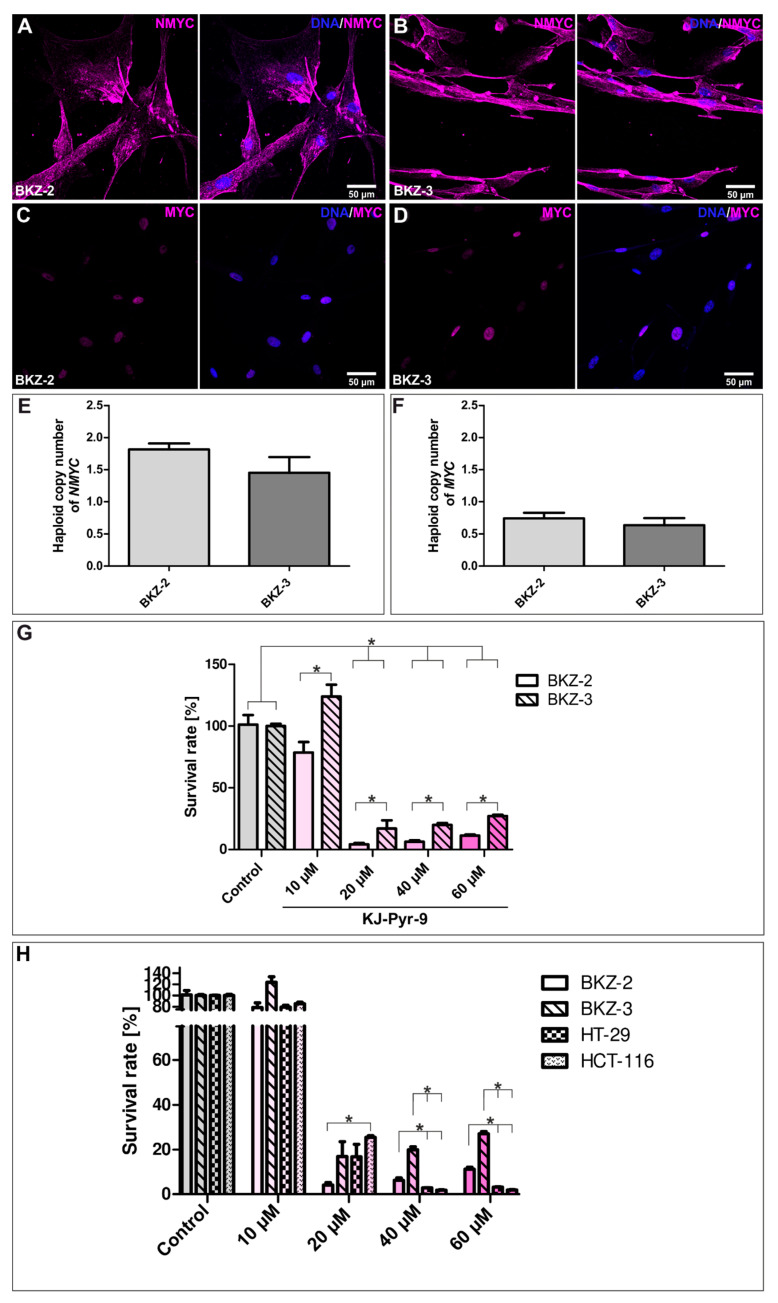
Inhibition of the myc proto-oncogene protein (MYC) and N-myc proto-oncogene protein (NMYC) significantly decreases the survival rate of BKZ-2 and BKZ-3 cells. Immunocytochemical staining revealed a strong expression of the oncogene (**A**/**B**) NMYC, as well as a nuclear expression of the oncogene (**C**/**D**) MYC, in both BKZ-2 and BKZ-3 on protein level. Evaluation of the haploid copy number of the two oncogenes, demonstrated a two-fold increase of the haploid copy number of (**E**) *NMYC*, but a normal haploid copy number for (**F**) *MYC* within both cell lines. To investigate the influence of the MYC/NMYC inhibitor KJ-Pyr-9 on the proliferation, 3000 cells per 100 μL cancer stem cell medium were cultured in a 96 well for 120 h with the inhibitor or dimethylsulfoxide and 10% fetal calf serum. Afterwards, metabolism was measured using Orangu^TM^ (Cell Guidance Systems, Cambridge, UK) and cell count was determined by using a standard curve. (**G**) Normalized survival rate was quantified and significantly decreased after exposure to values greater than 20 μM of KJ-Pyr-9 in comparison to the control for BKZ-2 and BKZ-3. Further comparisons between the two cell lines displayed a significant decrease of the survival rate of BKZ-2 in comparison to BKZ-3 for all inhibitor concentrations. (**H**) Although comparison of survival rates after KJ-Pyr-9-treatment showed significantly higher survival of HCT-116 when compared to BKZ-2 after 20 μM K-Pyr-9, cell survival of BKZ-2 and BKZ-3 was significantly improved in comparison to HT-29 and HCT-116 after treatment with inhibitor concentrations over 40 μM. Non-parametric Mann-Whitney-test (*p* ≤ 0.05). *n* = 3, * *p* ≤ 0.05. Mean ± SEM (standard error of the mean).

MYC and NMYC were reported to play an important role for plasticity, proliferation and apoptosis in various entities of malignancies. To investigate a possible correlation of ATRA resistance and MYC/NMYC, we analyzed BKZ-2 and BKZ-3 regarding MYC/NMYC expression levels. First, we determined protein expression levels in BKZ-2 and BKZ-3 cells using immunocytochemistry, revealing the expression of NMYC and MYC in both. Although both oncogenes were expressed, NMYC was mainly detected cytosolic contrary to MYC, which was detected in the nuclei of BKZ-2 and BKZ-3 (Figure 11A–D). 

Further, relative haploid normalized gain in gene copy numbers of *NMYC* and *MYC* was investigated for BKZ-2 and BKZ-3. Calculation of relative haploid copy number of *NMYC* displayed a 1.82 (±0.094)-fold amplification for BKZ-2 and a 1.45 (±0.24)-fold amplification for BKZ-3, indicating a duplication of the *NMYC* gene in both cell lines (Figure 11E). No amplification was detected for relative haploid gene copy number of *MYC*, as BKZ-2 represents a relative haploid copy number of 0.74 (±0.089) and BKZ-3 0.63 (±0.11) (Figure 11F). Thus, no differences in MYC and NMYC expression could be detected for BKZ-2 and BKZ-3, suggesting additional molecular pathways involved in ATRA mediated differentiation.

**Figure 12:**
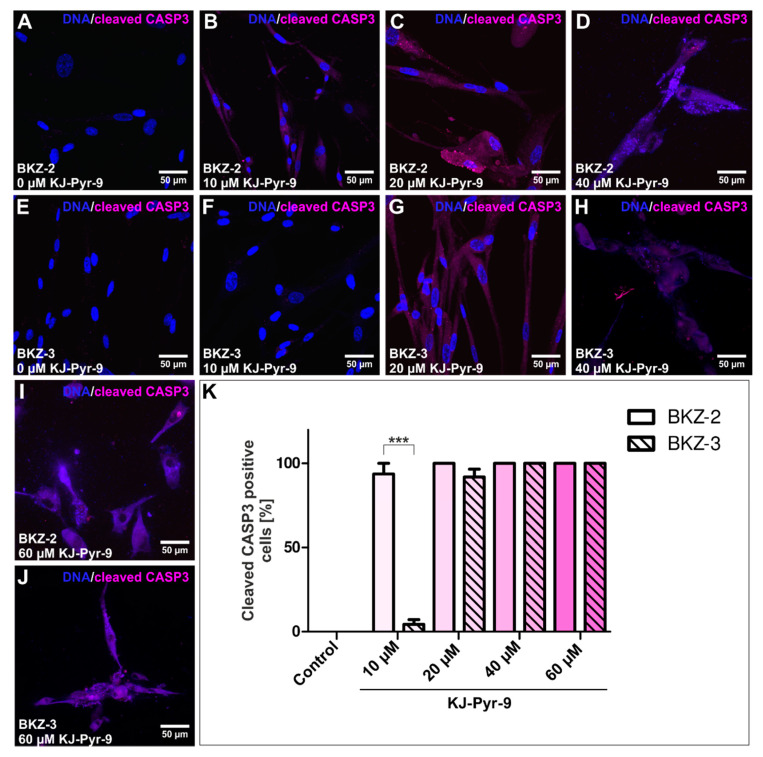
Myc proto-oncogene (MYC)/N-myc proto-oncogene (NMYC) inhibitor KJ-Pyr-9 induces apoptosis of BKZ-2 and BKZ-3. Representative images of immunocytochemical staining for cleaved caspase 3 (CASP3) after KJ-Pyr-9-treatment of (**A**–**E**) BKZ-2 and (**F**–**J**) BKZ-3. (**K**) Quantification of the percentage of cleaved CASP3 positive cells after the addition of 10 μM KJ-Pyr-9 revealed a significantly higher amount for BKZ-2 with about 94% in comparison to BKZ-3 with about 4%. However, concentrations higher than 40 μM lead to 100% cleaved CASP3 positive cells for BKZ-2 and BKZ-3. Student’s *t*-test (*p* ≤ 0.05). *n* = 3, *** *p* ≤ 0.001. Mean ± SEM (standard error of the mean).

Previously, KJ-Pyr-9 as inhibitor of both MYC and NMYC showed proliferation inhibiting effects on various tumor cell lines. Accordingly, we investigated this small molecule concerning its effect on survival of BKZ-2 and BKZ-3 cells. After 120 h co-incubation with the inhibitor, survival rate of both cell lines revealed a significant decrease (*p* ≤ 0.05) with increasing concentrations of KJ-Pyr-9 over 20 μM respectively. Moreover, discrepancies in the survival rates between both cell lines were detected, represented by significantly higher (*p* ≤ 0.05) survival rates for BKZ-3 in comparison to BKZ-2 after treatment with KJ-Pyr-9 independent on inhibitor concentrations (Figure 11G). Normalized survival rate after stimulation with 10 μM KJ-Pyr-9 was 78.59% (±8.53) for BKZ-2 and 123.9% (±9.62) for BKZ-3 and with 20 μM KJ-Pyr-9 4.12% (±1.09) for BKZ-2 and 16.89% (±6.61) for BKZ-3. Cultivation with 40 μM KJ-Pyr-9 led to a survival rate of 6.19% (±1.14) for BKZ-2 and 19.85% (±1.42) for BKZ-3 and with 60 μM KJ-Pyr-9 to a survival of 11.18% (±0.92) for BKZ-2 and 27.11% (±0.97) for BKZ-3. This significantly higher survival rate of BKZ-3 in contrast to BKZ-2 possibly demonstrates a higher MYC/NMYC inhibitor tolerance of BKZ-3, suggesting a more MYC-independent growth behavior in comparison to BKZ-2. This result stands in line with the here presented inefficient differentiation of BKZ-3 using ATRA, which is also known to target MYC/NMYC activity. Comparison of survival rate of BKZ-2 and BKZ-3 with the two colon carcinoma cell lines HT-29 and HCT-116 after KJ-Pyr-9-treatment revealed a significantly higher (*p* ≤ 0.05) survival of BKZ-2 and BKZ-3 subsequent to treatment with 40 μM and 60 μM (Figure 11H). Survival rates following the addition of 40 μM KJ-Pyr-9 were 6.19% (±1.14) for BKZ-2, 19.85% (±1.42) for BKZ-3, 2.83 (±0.12) for HT-29 and 1.78 (±0.27) and after 60 μM 11.18% (±0.92) for BKZ-2, 27.11% (±0.97) for BKZ-3, 3.18 (±0.16) for HT-29, and 1.93 (±0.14) for HCT-116. To address whether the reduction in survival is caused by regulation of proliferation or apoptosis BKZ-2 and BKZ-3 cells were treated with KJ-Pyr-9 as described before, followed by immunocytochemical staining for cleaved caspase 3 to determine the number of apoptotic cells (Figure 12A–J). Quantification of cleaved caspase 3 staining displayed a significantly higher (*p* ≤ 0.001) amount of apoptotic BKZ-2 cells with 93.65 (±6.35) after 10 μM KJ-Pyr-9 in comparison to BKZ-3 with 4.32 (±2.78). In accordance with that, BKZ-3 displayed a trend for less cleaved caspase 3 positive cells after 20 μM of KJ-Pyr-9 with 91.89 (±4.62) in comparison to BKZ-2 with 100% apoptotic cells. However, BKZ-2 seem to be more sensitive for KJ-Pyr-9 induced apoptosis, BKZ-2 and BKZ-3 showed 100% cleaved caspase 3 positivity for KJ-Pyr-9-incubation with concentrations greater than 40 μM (Figure 12K).

## 3. Discussion

In this study, we present the establishment of two primary human colorectal cancer cell lines that contain a subpopulation with potential stem-like properties. One very rare case of a GEP-NEC of the rectum is named BKZ-2 and one colorectal AC is referred to as BKZ-3. We characterized stemness-like properties by high CD133 and CD44 expression, relatively slow proliferation rates, and high number of formed spheres in the in vitro model systems, providing the basis for further exploration of patho-mechanisms in order to develop therapeutical strategies. As initial steps to that respect, we further tested for the impact and role of ATRA and the MYC/NMYC inhibitor KJ-Pyr-9 in these cells, as both are interfering with MYC/NMYC signaling in CCSC. However, some limitations of this study should be noted, as identification of the subpopulation with stem cell-like properties was only done by in vitro not in vivo experiments. 

CCSC are defined with a group of cell surface markers such as CD44, CD133, EPCAM and LGR5 [32,33], which all were tested positive in BKZ-2 and BKZ-3 cells on transcriptional and/or on protein level. CD133 is a robust biomarker to identify primary CSC and can be proposed as a prognostic marker of CRC patients. Moreover, it is reported to be expressed in poorly-differentiated NECs and well-differentiated NETs of the digestive tract [34] and was demonstrated to be expressed in CCSC-lines [35]. It is the most well-known marker for the isolation and investigation of CSC in different types of cancer [36,37,38] and known to be responsible for radio- and chemotherapy-resistance in CCSC [39,40]. However, it seems to be more reliable to use CD133 in combination with other markers, such as CD44 as target biomarkers for the isolation of CCSC in both cell lines and primary tumor cell populations as done in our study [6]. CSC-marker CD44 is known to mediate cancer cell survival, proliferation, and motility, as well as the modulation of tumor microenvironment [36,41,42], indicating CD133 and CD44 as an excellent marker set to validate a CCSC-like-phenotype. Next to the protein expression of CD133 and CD44, class VI intermediate filament protein Nestin was shown to be expressed in both cell lines, further confirming potential stem-like properties of BKZ-2 and BKZ-3 as Nestin is commonly accepted to play a role in CSC-phenotypes, particularly regarding the capacity for self-renewal [43]. Additionally, in vitro tumorigenicity, as proven here for BKZ-2 and BKZ-3 utilizing sphere formation assays and the higher number of formed spheres of BKZ-2 and BKZ-3 in comparison to established colon carcinoma cell lines HT-29 and HCT-116, underline the existence of a subpopulation with potential stem-like characteristics [15]. Moreover, sphere formation seems to be biologically reliable as these in vitro testing modalities demonstrated uniformly results corresponding with murine in vivo xenograft models if tested with the same cancer cells including NEC-cell lines [9,44,45]. Further analysis of the proliferation and comparison of population doubling times of BKZ-2 and BKZ-3 with established colon carcinoma cell lines HT-29 and HCT-116, respectively, showed significantly higher population doubling times for BKZ-2 and BKZ-3. This may be due to the fact, that CSC have the ability to become more quiescent with slow proliferation rates to escape from chemotherapy. These slow-cycling cells are tumorigenic and more resistant to traditional chemotherapies than rapidly dividing cells [46,47]. Analysis of *BRAF* mutation status showed no mutation for codons 600, 464, 466, and 469 in both parental tumor tissue and cell populations. Concerning *BRAF* mutation in CRC a retrospective study showed that patients whose tumors had microsatellite stability with mutant *BRAF* demonstrated significantly reduced overall survival [48]. However, only 6–18% of CRC patients harbor a *BRAF* mutation [48,49,50].

Quantitative PCR analysis displayed *LGR5* expression in both BKZ-2 and BKZ-3 cells. Nevertheless, their expression levels were significantly lower in comparison to HDF. LGR5 is well-established as a marker of native intestinal stem cells [51] as well as CCSC [52] and plays an active role in pathogenesis of CRC [53]. Certainly, Fumagalli and colleagues observed most disseminated CRC cells in circulation to be LGR5 negative. Latter cells formed distant metastases in which CSC appeared LGR5 positive [54]. This plasticity of CCSC may explain the relatively low level of *LGR5*, as BKZ-2 and BKZ-3 also revealed an EMT-phenotype reflected by a high level of SLUG. Positivity in BKZ-2 and BKZ-3 cells for the 40 kDa single transmembrane protein *EPCAM* encoded by tumor-associated calcium signal transducer-1 gene [55] was relevant as epithelial adhesion molecule playing a role in carcinogenesis of epithelial cells by activating expression of proto-oncogenes *MYC* and *CYCLIN A/E* [56]. Further, it was demonstrated to correlate with the level of malignancy in foregut-NET of the pancreas [57]. It was also shown to be involved in regulation of intercellular adhesion-mediated signal transduction, cell migration, proliferation and differentiation [55]. Significantly higher expression of *SOX2* was observed in primary CRC tissues and metastatic tumor tissues compared to paratumoral tissues with 80% of the analyzed primary tumor samples [58]. Further, in a CRC-derived CSC-line (SW620) [59], SOX2 was associated with cell migration, invasion, colony formation and tumorigenesis as well as with spherogenicity and chemoresistance [58], representing SOX2 as a CSC-marker. Interestingly, a knock-down of SOX2 in SW620 cells induced a mesenchymal–epithelial transition process, reducing the migration and invasion capabilities of cells [60], and suggesting a link between EMT and stemness. *SOX2* as well as CSC-marker *OCT4* were expressed in BKZ-2 and BKZ-3. However, *OCT4* expression was also prominent in HDF, which is not unexpected as fibroblasts are able to express *OCT4* under different cell culture microenvironmental conditions [61]. Expression of *CD133*, *CD44*, *EPCAM*, *SOX2,* and *OCT4* substantiates that BKZ-2 and BKZ-3 include a subpopulation with potential stem-like properties. Moreover, differences in messenger RNA (mRNA) expression levels of *CD133*, *CD44*, *LGR5,* and *SOX2* between BKZ-2 and BKZ-3 possibly demonstrate a balance between different CSC-markers necessary for CSC maintenance. 

Histopathological analysis of Synaptophysin as a neuroendocrine marker within the tumor tissue revealed expression only within NEC. However, immunocytochemically both, BKZ-2 and BKZ-3 were demonstrated to be positive for Synaptophysin. This finding emphasizes that Synaptophysin not only plays a role as neuroendocrine marker [62], but also as marker for stemness, as recently reported by us [63]. Quantification of Synaptophysin in nuclei revealed significantly more highly positive nuclei for BKZ-2 in comparison to BKZ-3, possibly reflecting the neuroendocrine origin or a potential more stem cell-like phenotype. Further investigation on neuronal and neural-crest related protein expressions displayed a strong expression of S100A1 for BKZ-2 and BKZ-3, which was shown to be negatively associated with the frequency of lymph node metastasis and level of dedifferentiation in ovarian cancer, enhancing the ovarian cancer cell proliferation and migration [64]. Further, S100A9 was shown to be required for the proliferation of CRC spheroids upon mammalian target of rapamycin complex 1 signaling [65], suggesting a role of S100 family in stemness of CRC cells. Moreover, the neuronal marker vGLUT2 [66], as well as the dopaminergic marker dopamine were detected in BKZ-2 and BKZ-3, highlighting the neuroendocrine origin of BKZ-2 and suggesting a neuronal differentiation potential for BKZ-3, too [67]. TH protein, which is a rate-limiting enzyme of dopamine synthesis [68], and thus a marker for dopaminergic neurons, was expressed predominantly nuclear, but also cytosolic in BKZ-2 cells. Moreover, it could be detected in the nuclei of BKZ-3 cells, possibly reflecting the multipotent phenotype of a subpopulation with potential stem cell-like characteristics, since mature neurons would express TH predominantly cytosolic. 

There is increasing evidence that CSC lay the foundation for cancer invasiveness. A critical role of EMT and stemness for tumor plasticity and aggressiveness was discussed for NET variants of prostate cancer in more depth [69,70,71] and to some extent for pancreatic neoplasms [72]. Additionally, targeting cancer cells using ATRA-dependent differentiation therapy has been recently described for several solid cancer types including CRC [29] and has been clinically proven for acute promyelocytic leukemia. There are indications that ATRA suppresses proliferation, migration, CSC population size and sphere formation of cancer cell lines by reversing EMT. During EMT, the E-cadherin promoter is frequently repressed by specific transcriptional repressors, including *SNAIL*, *SLUG*, *ZEB1*, high mobility group AT-hook 2, and *TWIST*, making E-cadherin expression a valuable prognostic factor of CRC [73]. We even demonstrated for precursor lesions of CRC *SNAIL*-up- and e-cadherin-down-regulation, which was not found in normal colorectal tissue [74]. In this study, BKZ-2 expressed higher level of the EMT key transcription factors *TWIST*, *SLUG*, and *SNAIL* as well as SLUG on protein level in comparison to BKZ-3. Thus, BKZ-2 seem to harbor a more pronounced EMT-phenotype in comparison to BKZ-3. Cui and coworkers demonstrated that ATRA induced differentiation by decreasing invasion, proliferation and migration of the murine hepatocellular carcinoma cell lines hepa1-6. Moreover, they showed impaired in vitro liver function upon the reversal of EMT [75]. Those results stand in line with the study of Modarai and colleagues, in which ATRA-treatment of human CRC lines decreased proliferation, sphere formation and ALDH + CSC population size by inducing neuroendocrine differentiation [29]. Within the presented study, this phenomenon was also shown for the neuroendocrine-derived cell line BKZ-2, revealing a decreased sphere volume and reduction in total cell mass, which is probably caused by decreased proliferation. Further, BKZ-2 cells seem to demonstrate a more differentiated phenotype after ATRA-treatment. However, analysis of the ATRA effect on apoptosis revealed no increase in apoptotic cells after ATRA-stimulation for BKZ-2 and BKZ-3. This suggests that ATRA suppresses proliferation in neuroendocrine-derived colorectal cancer cells with potential stem-like properties possibly by targeting an EMT-phenotype and inducing differentiation, as already shown for human CSC of head and neck squamous carcinoma [76], glioblastoma multiforme [77], and gastric cancer [78]. The AC-derived colorectal cancer cell line BKZ-3 exhibited lower mRNA levels of EMT transcription factors as well as SLUG high nuclei and behaved contrary subsequent to ATRA-treatment, as volume of spheres increased with no change in total cell mass. This possibly represents some sort of ATRA resistance for BKZ-3 cells, even if ATRA induced a shift in growth behavior for BKZ-3 cells. There is growing evidence that mechanisms inducing the degradation of retinoic acid receptor β [79] or cytoplasmic retinoid X receptor α play crucial roles in the ATRA resistance of colonic AC cell lines [80]. A similar downregulation of retinoic acid receptors could explain the opposed effect in BKZ-3 cells compared to BKZ-2, however the actual molecular pathway behind this still needs to be clarified. The CSC-marker ALDH [81] is a key enzyme in retinoid acid signaling and was shown to be targeted by ATRA in CRC cell lines [29]. Analysis of ALDH activity demonstrated a slightly higher proportion of BKZ-3 cells with high ALDH activity of about 26% in comparison to BKZ-2 with about 8% of highly active cells. However, Khorrami and coworkers have shown that HT-29-derived colonospheres with low ALDH activity demonstrate increased tumorigenic potential and stemness properties, reflecting the need for a set of CSC-marker and CSC plasticity. Even though it was presented that ATRA treatment decreased colony and sphere formation capacities only in an ALDH-high not in ALDH-low subpopulation of a human ovarian cancer cell line [82], the mechanism behind the higher sensitivity of BKZ-2 in comparison to BKZ-3 cannot be explained solely by ALDH activity.

There is evidence that differentiation therapy using ATRA involves MYC/NMYC regulation in cancer cells. This was shown by Farrell and coworkers, who validated that peptidyl-prolyl cis-trans isomerase Pin1 (PIN1) regulates MYC activity by acting on MYC degradation and activation [83]. ATRA acts as inhibitor of PIN1, reducing for example in vivo growth of triple-negative breast cancer xenografts by the degradation of PIN1 protein [84]. This stands in line with results concerning hepatocellular carcinoma xenografts using slow-release poly L-lactic acid microparticle containing ATRA [85]. Less sensitivity of BKZ-3 for ATRA-treatment was not reflected by differences in MYC-family member protein expressions but may be due to dysregulation in PIN1 signaling, leading to less inhibitory effects of ATRA on MYC activity. MYC-family proteins function as potent transcription factors that organize multiple cellular processes, including adhesion, proliferation, survival, and differentiation. Thus, MYC expression is frequently enhanced in cancer leading to elevated MYC RNA and protein expression [86,87]. Likewise, we demonstrated gene copy number gain for *NMYC* in both colorectal cancer cell lines that comprise a subpopulation of cells with potential stem-like properties. Gain of gene copy numbers of members of the *MYC*-family is reported to be an independent factor for poor prognosis in consecutive CRC patients and in the stage II–III subgroups [88]. Accordingly, neuroendocrine prostate cancer tumors, associated with aggressive disease and poor prognosis, are partly driven by aberrant expression of NMYC. NMYC overexpression and its subsequent deoxyribonucleic acid (DNA) binding induce epigenomic and transcriptomic reprogramming, resulting in a castration-resistant, promoting lineage-plasticity [21]. Further, NMYC overexpression was associated with highly proliferative, invasive prostate cancer with neuroendocrine features and was associated with an induction of EMT genes and poor outcome [89]. In accordance to that, *NMYC* copy number gain of BKZ-2 and BKZ-3 correlated with the expression of EMT genes and stem-like characteristics. Moreover, *NMYC* copy number gain of BKZ-2 as a neuroendocrine-derived colorectal cancer cell population goes along with the known correlation of neuroendocrine neoplasms with NMYC derangement in prostate cancer. As NMYC was shown to drive transformation of human prostate epithelial cells to prostate AC and neuroendocrine prostate cancer [90], BKZ-3 may present a lineage plastic AC-derived colorectal cancer cell line with a subpopulation of cells with potential stem-like characteristics and the potential to gain neuroendocrine features. Even though a copy number gain was not detected for *MYC*, it was also expressed on protein level within BKZ-2 and BKZ-3 cells. Thus, probably playing a role in maintaining CCSC-like phenotype, too. It was demonstrated that MYC positively regulate check point inhibitor proteins leukocyte surface antigen CD47 and PDL1 via direct binding to their encoding gene promoters suppressing both the innate and the adaptive immune response while favoring tumor growth [91]. Further, immune checkpoint ligand PDL1 is upregulated in EMT-activated human breast cancer cells [92] and was shown to be more highly expressed in metastatic CRC than in primary CRC [93], suggesting its correlation with stem cell characteristics. Intriguingly, EMT and immune checkpoint proteins maybe related to each other as EGFR activation induces EMT and PDL1 expression in cancer. Moreover, MYC is required for EGFR-mediated PDL1 upregulation but not EMT [94]. Further, in vitro experiments on CRC-derived tumor cell lines provided evidence that PDL2 is involved in tumor cell invasion [95]. Giving these facts, the higher level of EMT genes, SLUG protein and immune checkpoint ligands of BKZ-2 are probably associated with each other and may reflect a more invasive and aggressive phenotype of the isolated neuroendocrine-derived colorectal cancer cell line BKZ-2 in comparison to BKZ-3 cells. Accordingly, the difference in PDL1 expression was also detected in parental tumor tissue of the two cell lines with 2% of PDL1 positive vital tumor cells shown in BKZ-2 originating tissue and 0% in BKZ-3, respectively. Further analysis on CPS displayed a value of 2% for parental tumor tissue of BKZ-2 and a CPS of 3 for tissue donating BKZ-3 cells. Thus, a slight correlation between higher PDL1 expression in tumor cells and less PDL1 positive immune cell infiltration may be assumed. 

MYC/NMYC inhibition decreases the survival rates of both BKZ-2 and BKZ-3 cells. However, survival rate was significantly higher for BKZ-3 cells, further reflecting a protective mechanism against MYC/NMYC inhibitory molecules or rather a more MYC/NMYC independent growth of BKZ-3. Moreover, BKZ-2 and BKZ-3 seem to be more resistant to KJ-Pyr-9 in comparison to HT-29 and HCT-116, with BKZ-3 even revealing higher survival after 10 μM KJ-Pyr-9 treatment. This may be due to lower expression levels of the CSC-markers CD133 and CD44 in HT-29 and HCT-116, which stands in line with a study of Zhang and coworkers demonstrating a CD133 high expressing subpopulation of HT-29 cells paralleled by higher levels of MYC. Further, MYC silencing led to higher chemo sensitivity [18]. The KJ-Pyr-9 inhibitor used here as a small molecule antagonist of the protein–protein interaction of MYC and NMYC with myc-associated factor X (MAX) was reported to inhibit dimerization of these two molecules. Reduced survival rates were demonstrated subsequent to KJ-Pyr-9-treatment in vitro on various tumor cell lines with concentrations of 5 to 20 μM as well as in vivo in a human to mouse tumor xenograft model with a dosage of 10 mg/kg body weight [96], which was here presented for BKZ-2 and BKZ-3, too. Moreover, KJ-Pyr-9-treatment led to high percentages of apoptotic cells, revealing that its influence on survival is based on apoptosis. Interestingly, BKZ-2 cells showed significantly higher sensitivity in comparison to BKZ-3 when treated with 10 μM. Even though BKZ-3 was less sensitive for MYC/NMYC inhibition in comparison to BKZ-2, both colorectal cancer cell lines revealed significant reductions of survival rates due to induced apoptosis upon MYC/NMYC inhibition. Consequently, the usage of inhibitors of the protein–protein interaction of MYC and NMYC with MAX is a possible treatment strategy for both neuroendocrine- and AC-derived colorectal cancer cell lines that harbor a subpopulation with potential stem-like properties. 

## 4. Materials and Methods 

### 4.1. Patients Clinical Characterisation and Oncological Treatment

Patient 1 (donor of BKZ-2 cell line) was a 79-year-old Caucasian male when admitted to the emergency room of the Protestant Hospital of Bethel Foundation (Bielefeld, Germany) with abdominal pain and clinical signs of an obstructive ileus. Computerized tomography (CT)-scan revealed a malignant tumorous lesion of the upper third of the rectum with consecutive massive dilated upstream colon (Figure 1A). Flexible rectal endoscopy confirmed a luminal subtotally occluding neoplastic lesion (Figure 1B). Following the introduction of a self-expanding metallic endo-prosthetic stent, a passage was reestablished (Figure 1C). Staging revealed multiple synchronic hepatic metastases of all segments (Figure 1D). Patient 1 was scheduled for robotic deep rectal resection with primary, circular stapler performed descendo-rectostomy. Following national guidelines and the recommendation of the local oncological board, a systemic chemotherapy with carboplatin and Etoposid was initiated and further switched to cisplatin and Etoposid due to hepatic progress. Patient 2 (donor of BKZ-3 cell line) was a 64 year old Caucasian male when admitted to the emergency room of the Protestant Hospital of Bethel Foundation (Bielefeld, Germany) with signs of sub-total ileus, abdominal crampy pain and a history of recurring nausea and vomitus paralleled by weight loss of 15 kg in the last three months prior admission. The patient presented a bloated abdomen without peritonism and scarce peristaltic of the bowel. CT-scan revealed a mass of the splenic flexure of the left colon suspicious for malignancy with distension of the pre-stenotic bowel (Figure 1E). Staging revealed no evidence of extra colonic malignant lesions. The patient was urgently scheduled for an open left hemicolectomy with worsening signs of mechanical bowel obstruction. 

Isolation of genomic DNA of parental tumor tissue for *BRAF* mutation analysis was performed using automatically DNA-extraction with Maxwell^®^ RSC DNA FFPE Kit and the Maxwell^®^ RSC instrument (Promega, Walldorf, Germany) according to the manufacturers guidelines in the Institute of Pathology KRH Hospital Nordstadt (Hannover, Germany). Sequencing of isolated genomic DNA of parental tissue and genomic DNA of isolated cell lines (as described below) was performed using DTCS Quick Start Kit (Beckman Coulter Life Sciences, Indianapolis, IN, USA) and GenomeLab GeXP™ Genetic Analysis System (Beckman Coulter Life Sciences) according to the manufacturers guidelines. 

Informed consent according to local and international guidelines was signed by both patients. All further experimental procedures were ethically approved (Ethics committee Münster, Germany, 2017-522-f-S).

### 4.2. Colorectal Cancer Cell Line Establishment and Cell Culture

To obtain primary tumor material for cell culture, a cubic sample measuring 5 mm was collected from each tumor type. The sample was transferred to ice-cold Dulbecco’s Phosphate Buffered Saline (PBS; Sigma Aldrich, Munich, Germany) supplemented with antibiotics penicillin/streptomycin (500 μg/mL; Sigma Aldrich), gentamicin (100 μg/mL; Ratiopharm, Ulm, Germany) and metronidazole (5 μg/mL; B.Braun, Melsungen, Germany), as well as the antimycotic amphotericin B (12.5 μg/mL; Sigma Aldrich) to control colic microbiom contamination. For the isolation of the cells, the specimen was washed ten times with ice-cold PBS, mechanically disintegrated in 2–5 mm pieces followed by enzymatical digestion with collagenase for 2 h at 37 °C as described previously [63]. One half of the minced tissue was used to cultivate spheres in Dulbecco’s Modified Eagle’s medium/Ham’s F-12 (Sigma Aldrich) with the addition of 2 mM L-Glutamin (Sigma Aldrich), penicillin/streptomycin (100 μg/mL), gentamicin (20 μg/mL), metronidazole (1 μg/mL), amphotericin B (2.5 μg/mL), EGF (20 ng/mL; Miltenyi Biotec, Bergisch Gladbach, Germany), FGF-2 (40 ng/mL; Miltenyi Biotec) and B27 supplement (Gibco, Thermo Fisher Scientific, Bremen, Germany) in low adhesion T25 tissue culture flasks. The other half of the tissue was used to grow adherent cells, where the cells were cultivated on gelatin from bovine skin (type B; Sigma Aldrich) coated culture dishes in the medium described above supplemented with 10% FCS (Sigma Aldrich). 

Colon adenocarcinoma cell line HT-29 (DSMZ-German Collection of Microorganisms and Cell Cultures, Braunschweig, Germany) and colon carcinoma cell line HCT-116 (DSMZ-German Collection of Microorganisms and Cell Cultures) were maintained in McCoy’s 5A medium (Sigma Aldrich) with 10% FCS, 2 mM L-Glutamin, and penicillin/streptomycin (100 μg/mL). Adult HDF (Genlantis, San Diego, CA, USA) were a kind gift from Dr. Isabel Faust, Institute for Laboratory and Transfusion Medicine, Heart and Diabetes Centre NRW (Bad Oeynhausen, Germany) and were cultured in Dulbecco’s Modified Eagle’s Medium-high glucose (Sigma Aldrich) with 10% FCS, 2 mM L-Glutamin, and penicillin/streptomycin (100 μg/mL). All cells were cultured at 37 °C and 5% CO2 in a humidified incubator.

Population doubling times were determined using the Orangu^TM^ Cell Counting Solution and were performed at least six times per cell population according to the manufacturers guidelines. For this 1000, 2500, 5000, 7500, and 10,000 cells per 100 μL CSC medium containing 10% FCS were seeded in a 96 well and used as a standard curve. To calculate the population doubling time 3000 cells per 100 μL CSC medium containing 10% FCS were seeded in a 96 well and cultured for 72 h, followed by the measurement of cell viability and quantification of cell count using the appropriate standard curve. Growth rates and population doubling times were determined by the following equations:

(1) $$growth\;rate=\frac{\ln\left(xt\right)-\ln\left(x0\right)}{t-t0}$$

(2) $$population\;doubling\;time=\frac{\ln\left(2\right)}{growth\;rate}$$

### 4.3. Immunocytochemistry and Immunohistochemistry

For immunocytochemical cell staining, BKZ-2, BKZ-3, HT-29, HCT-116, and HDF were pre-cultured as an explant culture as described above. After harvesting, 1.5 × 10^4^ cells per 500 μL CSC medium supplemented with 10% FCS were planted on top of etched cover slips. After 2–3 days of cultivation, cells were fixed with 4% phosphate-buffered paraformaldehyde (lab-made) for 15 min at room temperature (RT) followed by three washing steps with 1 × PBS. Cells were blocked and permeabilized using 0.02% Triton-X 100 (Sigma Aldrich) with 5% appropriate serum (Dianova, Hamburg, Germany) or 1% bovine serum albumin (Sigma-Aldrich) for 30 min at RT. Afterwards, cells were incubated with primary antibodies for 1 h at RT. Antibodies used were anti-CD44 (1:400; 156-3C11; Cell Signaling, Frankfurt am Main, Germany), anti-CD133 (1:100; NB120-16518; NovusBio, Bio-Techne, Wiesbaden-Nordenstadt, Germany), anti-Nestin (1:200; MAB5326; Millipore, Merck, Darmstadt, Germany), anti-Synaptophysin (1:250; MAB5258;Abcam, Berlin, Germany), anti-SLUG (1:100; C19G7; Cell Signaling), anti-S100 (1:400; Z 0311; DAKO, Agilent, Santa Clara, CA, USA), anti-Dopamine (1:100; AB1225; Millipore, Merck), anti-vGLUT2 (1:50; MAB5504; Millipore, Merck), anti-TH (1:50; H-196; Santa Cruz, Heidelberg, Germany), anti-Cleaved Caspase-3 (1:400; Asp175/5A1E; Cell Signaling), anti-MYC (10 μg/mL; Y69; Abcam) and anti-NMYC (2,5 μg/mL; NCM II 100; Abcam). Secondary fluorochrome-conjugated antibodies (1:300; goat anti-mouse Alexa 555, goat anti-rabbit Alexa 555, goat anti-mouse Alexa 488; Life Technologies, Thermo Fisher Scientific) were incubated for 1 h at RT in the dark. Nuclear counterstaining was performed with 4′,6-diamidino-2-phenylindole (DAPI; 1 μg/mL; Sigma Aldrich) for 10 min at RT. Fluorescence imaging was performed using a confocal laser scanning microscope (LSM 780; Carl Zeiss, Jena, Germany) and analyzed using ZEN software from the same provider or Fiji ImageJ [97]. For the quantification of immunofluorescence stainings, at least five images per condition were analyzed. Percentages of SLUG and Synaptophysin high and low cells were quantified by the mean of the nuclear fluorescence intensity, with cells showing a nuclear mean fluorescence intensity over 15,000 clustered as SLUG and Synaptophysin high. CD133 and CD44 high, medium and low cells were determined by 10 randomly applied measurements for fluorescence intensity on at least five images per cell population. HDF were used as negative control and their background fluorescence was subtracted from each measurement. Cells were classified as CD133 high: ≥ 10,000, CD133 medium: < 10,000 ≥ 5000 and CD133 low: < 5000 ≥ 0. For CD44 expression, cells were classified in CD44 high: ≥ 2000, CD44 medium: < 2000 ≥ 1000 and CD44 low: < 1000 ≥ 0.

All immunohistochemical stainings, except for the staining for MYC and NMYC, were performed in the Institute of Pathology of KRH Hospital Nordstadt (Hannover, Germany) using the automated immunohistochemistry and in situ hybridization platform Dako Omnis (DAKO, Agilent) according to the manufacturer’s instructions. Antibodies used were: Anti-CD56 (123C3; DAKO, Agilent), anti-CK20 (K_s_20.8; DAKO, Agilent), anti-Ki67 (MIB-1; DAKO, Agilent), anti-Synaptophysin (DAK-SYNAP; DAKO, Agilent), anti-CDX2 (DAK-CDX2; DAKO, Agilent), anti-panCK (AE1/AE3; DAKO, Agilent), anti-PDL1 (22C3; DAKO, Agilent), anti-CK7 (OV-TL 12/30; DAKO, Agilent) and anti-SATB2 (EP281; Cell Marque, Rocklin, CA, USA). For visualization the EnVision FLEX, High pH (DAKO, Agilent) visualization system was used according to the manufacturers guidelines. Each scoring pathologist had specific training for the specific antibody and particular indication for testing as appropriate. Analysis of the expression of the immune checkpoint ligand PDL1 was done according to international standards [98].

(3) $$TPS=\frac{PDL1\;positive\;vital\;tumor\;cells}{PDL1\;positive+negative\;vital\;tumor\;cells}$$

(4) $$CPS=\frac{PDL1\;positive\;vital\;tumor\;cells+lymphocytes+macrophages}{PDL1\;positive+negative\;vital\;tumor\;cells}*100$$

(5) $$IC=\frac{PDL1\;positive\;lymphocytes+macrophages+dendritic\;cells+granulocytes}{tumor\;area}$$

IC is given as a score with the following values: 0 = 0–<1%, 1 = ≥1%–<5%, 2 = 5%–<10%, 3 = ≥10%. For immunocytochemical staining of MYC and NMYC paraffin-embedded sections were deparaffinized and rehydrated. For this, sections were washed two times for 10 min in xylol followed by 10 min 100% ethanol. Afterwards, sections were rehydrated by 5 min washes in 90% ethanol, followed by 80% ethanol and 70% ethanol. Epitope retrieval was performed by boiling the slides within 0.01 M citrate buffer, pH 6.0 (lab made) for 20 min. After cool down for at least 30 min at RT slides were washed two times with 0.02% Triton-X 100. Afterwards, slides were blocked and permeabilized using 0.02%Triton-X 100 with 10% appropriate serum (Dianova) and 1% bovine serum albumin for 2 h at RT. Anti-MYC (5 μg/mL; Y69; Abcam) and anti-NMYC (5 μg/mL; NCM II 100; Abcam) first antibodies were diluted in blocking solution and incubated over night at 4 °C. After three washing steps in PBS, secondary fluorochrome-conjugated antibodies (1:300; goat anti-mouse Alexa 555, goat anti-rabbit Alexa 555; Life Technologies, Thermo Fisher Scientific) were applied and incubated for 1 h at RT in the dark. Nuclear counterstaining was performed with DAPI (1 μg/mL) for 10 min at RT and fluorescence imaging was performed using a confocal laser scanning microscope (LSM 780; Carl Zeiss) and analyzed using Fiji ImageJ.

### 4.4. Sphere-Formation and ATRA-Treatments

To analyze the sphere-formation capacity of BKZ-2 and BKZ-3, cells were cultured in low adhesion culture plates in CSC medium without FCS with different concentrations of heparin. Cells were seeded in triplicates at 1 × 10^5^ cells per 1000 μL CSC medium. Heparin (Sigma Aldrich) was added in concentrations of 3 μg/mL, 4 μg/mL, 5 μg/mL or 0 μg/mL as a control. Cells were cultured for seven days in a humidified cell incubator at 37 °C and 5% CO_2_. The size of the spheres was quantified for each treatment condition at day 2, day 4, and day 7 after initial cell seeding. Therefore, five randomized images of each well were taken, and every sphere was measured in length and width using Fiji ImageJ. Then, the diameter of each sphere as mean of length and width was calculated. For the comparison of sphere formation capacity of BKZ-2, BKZ-3 with HT-29 and HCT-116 and for sphere-formation under the treatment of different concentrations of ATRA (Sigma Aldrich), cells were seeded at 5000 cells per 200 μL CSC medium containing 4 μg/ml heparin in a low adhesion 96 well-plate and cultured for five days. For the treatment, medium was supplemented with 1 μM ATRA, 5 μM ATRA, 10 μM ATRA or dimethylsulfoxide (DMSO) as a control in triplicates for each treatment condition. For quantification, two representative images of each well were taken and every sphere was measured in length and width using Fiji ImageJ, with the mean of both representing the diameter of each sphere. Volume of spheres was determined using the formula:

(6) $$V=\frac{4}{3}*\pi *{\left(\frac{sphere\;diameter}{2}\right)}^{2}$$

The number of spheres was calculated by counting each sphere of all six representative images for each condition. For total cell mass calculation, mean of sphere volume was multiplied with the mean of sphere number. According to Weiswald and colleagues, spheres with a diameter of ≥ 20 μm were considered for further evaluation [17]. For the analysis of the influence of ATRA on BKZ-2 and BKZ-3 in monolayer, 3000 cells per 100 μL CSC medium containing 10% FCS were seeded in a 0.1% gelatin coated 96 well-plate. Additionally, 1000, 2500, 5000, 7500, and 10,000 cells per 100 μL medium were seeded in a 96 well for a standard curve. After adherence of the cells, cell viability was measured for the standard curve and ATRA was applied as described above. After 5 days of treatment, cell viability was measured and cell count was quantified using the standard curve. Each treatment condition was performed in triplicates. For the immunocytochemical staining of cleaved caspase 3 after ATRA-treatment, 1.5 × 10^4^ cells per 500 μL CSC medium containing 10% FCS were seeded in a 24 well on top of etched cover slips. After adherence, cells were treated with different ATRA concentrations as well as DMSO as control for five days. Afterwards, cleaved caspase 3 immunocytochemical staining was performed as described above.

### 4.5. Real-Time PCR

RNA isolation of cultivated cell populations was done using the NucleoSpin^®^ RNA Plus kit (Machery-Nagel, Düren, Germany) according to the manufacturers instruction. Quality and concentration of RNA were assessed via Nanodrop ultraviolet spectrophotometry. Copy DNA (cDNA) synthesis was performed using 1 μg of RNA and the First Strand cDNA Synthesis Kit (Thermo Fisher Scientific). For the synthesis, random hexamer primers were used. qPCR were performed in triplicates using 2 × qPCR SyGreen Mix (PCR Biosystems, London, UK), according to the manufacturer’s instructions, and assayed with the Eco48 (PCRmax, Stone, Staffordshire, UK). Used primers (Sigma Aldrich) are listed in Table 1. Isolation of the genomic DNA for the analysis of gene copy number of *NMYC* and *MYC* was done using QUIamp^®^ DNA Mini Kit (Qiagen, Hilden, Germany) according to the manufacturer’s guidelines. Quantification of gene copy number was performed in triplicates using Platinum SYBR Green qPCR Super-Mix UDG (Invitrogen, Thermo Fisher Scientific), according to the manufacturers guidelines, and assayed with a Rotor Gene 6000 (Qiagen). Each gene assay included: (1) a no-template control, (2) 10 ng of calibrator human genomic DNA of MSCs (Lonza, Basel, Switzerland), and (3) 10 ng of tumor DNA. Haploid copy number was determined according to De Preter et al. [99].

### 4.6. MYC Inhibitor Treatment

To analyze the influence of the proto-oncogenes *MYC* and *NMYC*, cells were treated with different concentrations of the MYC and NMYC inhibitor KJ-Pyr-9 (Merck) and cell viability assays using Orangu^TM^ were performed in triplicates according to the manufacturers guidelines. For this, cells were seeded in 0.1% gelatin coated 96 well-plates in the amount of 1000, 2500, 5000, 7500 and 10,000 cells per 100 μL CSC medium containing 10% FCS for a standard curve and in the amount of 3000 cells per 100 μL CSC medium containing 10% FCS for the treatment. After adherence of the cells, cell viability was measured for the standard curve and treatment was started applying KJ-Pyr-9 in the concentrations of 10 μM, 20 μM, 40 μM, and 60 μM, as well as DMSO as control to the CSC medium supplemented with 10% FCS. After 5 days of treatment, cell viability was measured, and cell count was quantified using the standard curve. Survival rate was calculated by normalizing each cell count to the mean of controls for BKZ-2 and BKZ-3. For the immunocytochemical staining of cleaved caspase 3 after KJ-Pyr-9-treatment, 1.5 × 10^4^ cells per 500 μL CSC medium containing 10% FCS were seeded in a 24 well on top of etched cover slips. After adherence, cells were treated with 10 μM, 20 μM, 40 μM or 60 μM of KJ-Pyr-9 or DMSO as a control for five days. Afterwards, cleaved caspase 3 immunocytochemical staining was performed as described above.

### 4.7. Flow Cytometry

To determine the ALDH-activity, BKZ-2 and BKZ-3 were pre-cultured as an explant culture as described above. After harvesting, using trypsin, cells were analyzed with the ALDEFLUOR™ Kit (STEMCELL Technologies Inc., Vancouver, BC, Canada) according to the manufacturer’s instructions by flow cytometry on a Gallios flow-cytometer (Beckman Coulter Life Sciences). Dead cells were excluded by Propidium Iodide (Sigma Aldrich) co-staining and spectral overlap was compensated using samples of the K562 cell line (DSMZ-German Collection of Microorganisms and Cell Cultures), which also served as positive control. BKZ-2 and BKZ-3 cells were analyzed in parallel to yield comparable results.

### 4.8. Statistical Analysis

Data were raised at least in triplicates and statistically analyzed by using Prism V5.01 software (GraphPad Software, Inc., San Diego, CA, USA). Test for normality was performed by use of D’Agnostino & Pearson omnibus normality test. Student’s *t*-test, non-parametric Kruskal–Wallis analysis of variance (ANOVA) and Dunn’s Multiple Comparison post-hoc test or non-parametric Mann–Whitney-test were performed to assess differences between multiple groups. A significance value of *p* ≤ 0.05 was considered as statistically significant. The data are presented as the means ± standard error of the mean (SEM).

## 5. Conclusions

In summary, we isolated the two novel colorectal cancer cell lines BKZ-2 and BKZ-3, originating from a rare rectal neuroendocrine carcinoma and a colorectal AC, respectively, both containing a subpopulation with potential stem-like properties. Thus, BKZ-2 and BKZ-3 represent excellent new in vitro models to study colorectal cancer in cellular systems. Higher expression of CD133 and CD44 of both cell populations and a higher number of formed spheres in comparison to well-established colon carcinoma cell lines HT-29 and HCT-116 validated the existence of a subpopulation with potential stem cell-like phenotype. Moreover, initial characterization revealed a gene copy number gain of the proto-oncogene *NMYC* for both cell lines. Indirect inhibition of MYC/NMYC activity with ATRA led to decreased sphere volume and total cell mass for BKZ-2, although not for BKZ-3, which is probably caused by its effect on proliferation. Inhibition of the protein–protein interaction of MYC and NMYC with MAX utilizing KJ-Pyr-9 exhibited a significant reduction in the survival rate of both cell lines by the induction of apoptosis. Consequently, the blockage of these interactions may serve as a possible treatment strategy for colorectal cancer cell lines with increased *NMYC* copy number. However, differences in phenotype and the modulation of proliferation subsequent to ATRA-treatment, respectively, underline the complexity of signaling pathways involved in tumorigenesis and CSC maintenance of the different subclasses of colorectal cancer cells warranting further studies on this relevant topic.

## Figures and Tables

**Table 1:** Primer sequences for quantitative polymerase chain reaction.

Target	Sequence 5′-3′
CD44 antigen (*CD44*)	CTACAAGCACAATCCAGGCAA
Rev-*CD44*	GCATTGGATGGCTGGTATGA
Programed death ligand 1 (*PDL1*)	CCCAGTTCTGCGCAGCTT
Rev-*PDL1*	ACCGTGACAGTAAATGCGTTC
Programed death ligand 2 (*PDL2*)	TCCAACTTGGCTGCTTCACA
Rev-*PDL2*	CCACAGGTTCAGATAGCACTGT
Prominin-1 (*CD133*)	AACAGTTTGCCCCCAGGAAA
Rev-*CD133*	GAAGGACTCGTTGCTGGTGA
Epithelial cell adhesion molecule (*EPCAM*)	GCTGGCCGTAAACTGCTTTG
Rev-*EPCAM*	ACATTTGGCAGCCAGCTTTG
N-myc proto-oncogene (*NMYC*) *(genomic)*	CGCAAAAGCCACCTCTCATTA
Rev-*NMYC (genomic)*	TCCAGCAGATGCCACATAAGG
Octamer-binding transcription factor 4 (*OCT4*)	CGAAAGAGAAAGCGAACCAG
Rev-*OCT4*	GCCGGTTACAGAACCACACT
Myc proto-oncogene (*MYC*) *(genomic)*	AAAAGTGGGCGGCTGGATAC
Rev-*MYC (genomic)*	AGGGATGGGAGGAAACGCTA
SRY-box transcription factor 2 (*SOX2*)	GGCACTTTGCACTGGAACTT
Rev-*SOX2*	AGGCTGCTGGTTTTCCACTA
Twist family bHLH transcription factor 1 (*TWIST*)	GTCCGCAGTCTTACGAGGAG
Rev-*TWIST*	CCAGCTTGAGGGTCTGAATC
Snail family transcriptional repressor 1 (*SNAIL*)	CCCAATCGGAAGCCTAACTA
Rev-*SNAIL*	GGACAGAGTCCCAGATGAGC
Snail family transcriptional repressor 2 (*SLUG*)	TCGGACCCACACATTACCTT
Rev-*SLUG*	TTGGAGCAGTTTTTGCACTG
Actin beta	TCCCTGGAGAAGAGCTACGA
Rev-Actin beta	AGCACTGTGTTGGCGTACAG
Eukaryotic translation elongation factor 2 (*EEF2*)	AGGTCGGTTCTACGCCTTTG
Rev-*EEF2*	TTCCCACAAGGCACATCCTC
Syndecan 4 *(genomic)*	CAGGGTCTGGGAGCCAAGT
Rev-Syndecan 4 *(genomic)*	GCACAGTGCTGGACATTGACA
Glyceraldehyde-3-phosphate dehydrogenase *(genomic)*	AGACTGGCTCTTAAAAAGTGCAGG
Rev-Glyceraldehyde-3-phosphate dehydrogenase *(genomic)*	TGCTGTAGCCAAATTCGTTGTC

## Supplementary Materials

The following are available online at https://www.mdpi.com/2072-6694/12/9/2582/s1, Figure S1: Expression of neuronal and neural crest related proteins in BKZ-2 and BKZ-3 cells, Figure S2: Immunocytochemical staining of adult human dermal fibroblasts (HDF) as negative control for confocal laser scanning microscopy, Figure S3: BKZ-2 and BKZ-3 are highly positive for cancer stem cell-markers prominin-1 (CD133) and CD44 antigen (CD44), Figure S4: All-trans retinoic acid (ATRA)-treatment reduces proliferation of BKZ-2 cells, but does not affect BKZ-3.
