# Supplementary material for: Isolation and Characterization of Two Novel Colorectal Cancer Cell Lines, Containing a Subpopulation with Potential Stem-Like Properties: Treatment Options by MYC/NMYC Inhibition
Source: Cancers (Basel). 2020 Sep 10;12(9):2582. doi: 10.3390/cancers12092582 (PMC7564713; doi:10.3390/cancers12092582)
Supplement: Supplementary file 1 [file cancers-12-02582-s001.pdf]

Supplementary Materials: Isolation and Characterization of Two Novel Colorectal Cancer Cell Lines, Containing a Subpopulation with Potential Stem-Like Properties: Treatment Options by MYC/NMYC Inhibition

Jan Schulte am Esch, Beatrice Ariane Windmöller, Johannes Hanewinkel, Jonathan Storm, Christine Förster, Ludwig Wilkens, Martin Krüger, Barbara Kaltschmidt and Christian Kaltschmidt

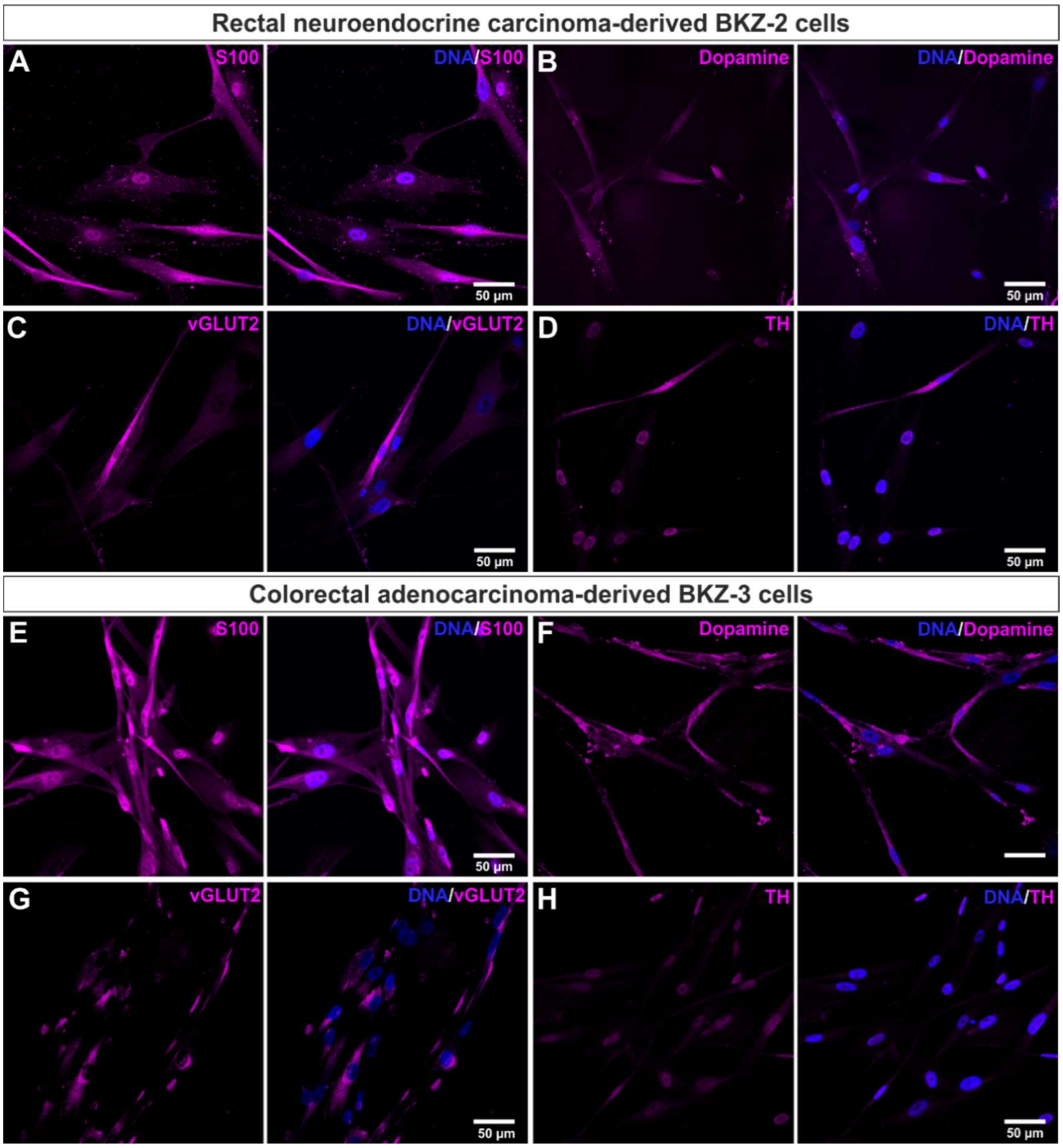

Figure S1. Expression of neuronal and neural crest related proteins in BKZ-2 and BKZ-3 cells. Immunocytochemical stainings revealed the expression of neural crest related (**A**) calcium binding protein S100 (S100), dopaminergic marker (**B**) Dopamine and neuronal markers (**C**) vesicular glutamate transporter 2 (vGLUT2) and (**D**) tyrosine hydroxylase (TH) in BKZ-2 cells. Immunocytochemical stainings of BKZ-3 displayed the expression of neural crest related protein **(E)** S100, dopaminergic marker (F) Dopamine and neuronal markers (**G**) vGLUT2 and neuronal marker (**H**) TH.

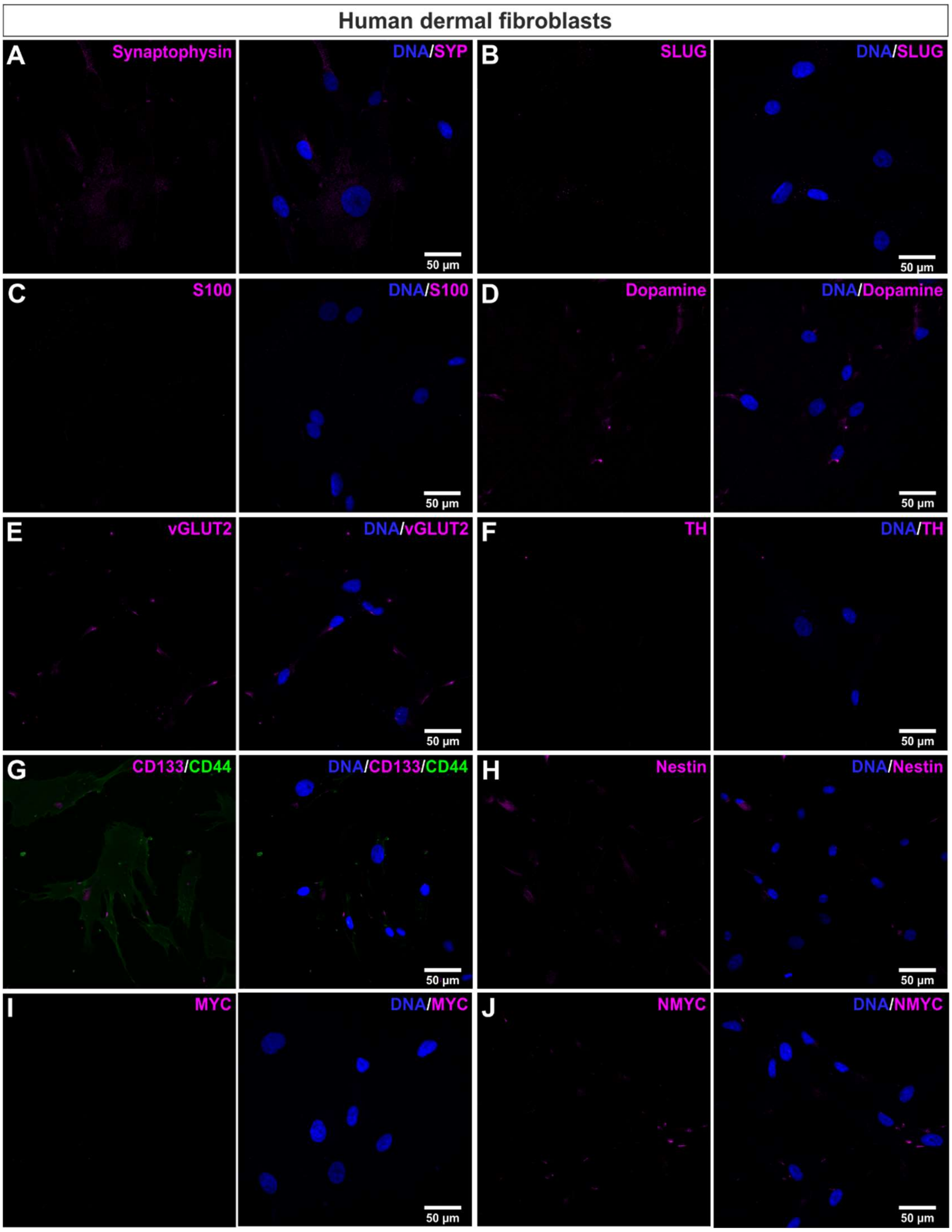

Figure S2. Immunocytochemical staining of adult human dermal fibroblasts (HDF) as negative control for confocal laser scanning microscopy. Immunocytochemical staining of HDF against (**A**) Synaptophysin (SYP), (**B**) Snail family transcriptional repressor 2 (SLUG), (**C**) calcium binding protein S100 (S100), (**D**) Dopamine, (**E**) vesicular glutamate transporter 2 (vGLUT2), (**F**) tyrosine hydroxylase (TH), (**G**) prominin-1 (CD133) / CD44 antigen (CD44), (**H**) Nestin, (**I**) myc proto-oncogene (MYC) and (**J**) N-myc proto-oncogene (NMYC) did not show any or only slight expression of these proteins.

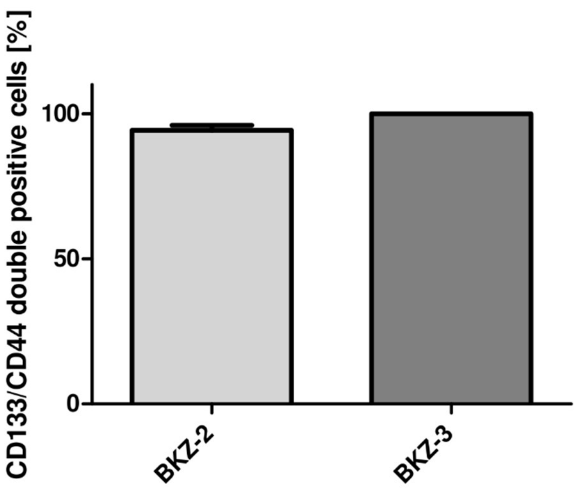

Figure S3. BKZ-2 and BKZ-3 are highly positive for cancer stem cell-markers prominin-1 (CD133) and CD44 antigen (CD44). Quantification of CD133 and CD44 expression revealed 94% of BKZ-2 and 100% of BKZ-3 double positive cells.

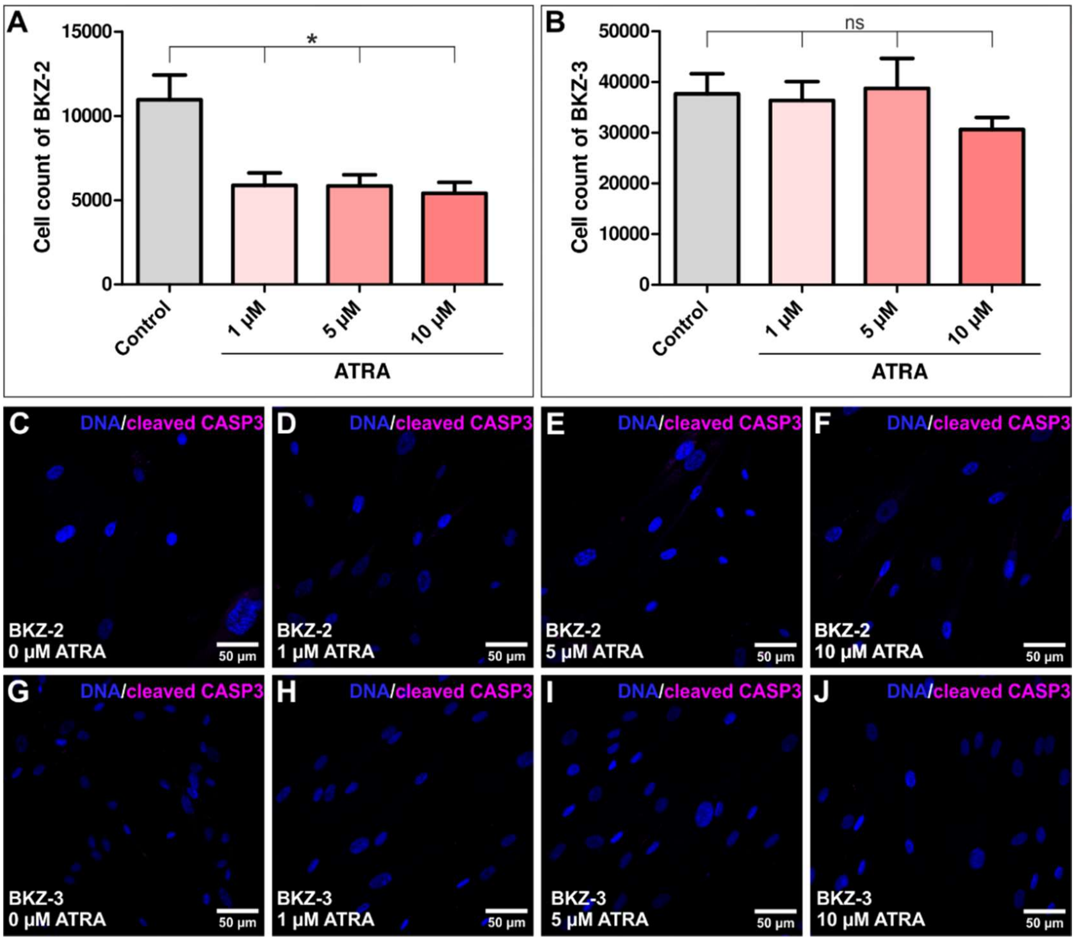

Figure S4. All-trans retinoic acid (ATRA)-treatment reduces proliferation of BKZ-2 cells, but does not affect BKZ-3. (**A**/**B**) Quantification showed a significant reduction in cell count for BKZ-2, but not for BKZ-3 upon ATRA-treatment. Immunocytochemical analysis of cleaved caspase 3 (CASP3) expression after ATRA-treatment did not show any expression for (**C**–**F**) BKZ-2 or (**G**–**J**) BKZ-3. Non-parametric Mann-Whitney-test ($p \leq 0.05$). $n = 3$, * $p \leq 0.05$, ns = not significant. Mean ± SEM (standard error of the mean).

© 2020 by the authors. Licensee MDPI, Basel, Switzerland. This article is an open access article distributed under the terms and conditions of the Creative Commons Attribution (CC BY) license (http://creativecommons.org/licenses/by/4.0/).
